# Bioinformatic and literature assessment of toxicity and allergenicity of a CRISPR-Cas9 engineered gene drive to control *Anopheles gambiae* the mosquito vector of human malaria

**DOI:** 10.1186/s12936-023-04665-5

**Published:** 2023-08-14

**Authors:** Alima Qureshi, John B. Connolly

**Affiliations:** Department of Life Sciences, Imperial College London, Silwood Park, Sunninghill, Ascot, UK

**Keywords:** Population suppression, Gene drive, Environmental risk assessment (ERA), Genetically modified mosquitoes (GMM), CRISPR, Cas9, DsRed, gRNA, Allergenicity, Toxicity

## Abstract

**Background:**

Population suppression gene drive is currently being evaluated, including via environmental risk assessment (ERA), for malaria vector control. One such gene drive involves the *dsxF*^*CRISPRh*^ transgene encoding (i) hCas9 endonuclease, (ii) T1 guide RNA (gRNA) targeting the *doublesex* locus, and (iii) DsRed fluorescent marker protein, in genetically-modified mosquitoes (GMMs). Problem formulation, the first stage of ERA, for environmental releases of *dsxF*^*CRISPRh*^ previously identified nine potential harms to the environment or health that could occur, should expressed products of the transgene cause allergenicity or toxicity.

**Methods:**

Amino acid sequences of hCas9 and DsRed were interrogated against those of toxins or allergens from NCBI, UniProt, COMPARE and AllergenOnline bioinformatic databases and the gRNA was compared with microRNAs from the miRBase database for potential impacts on gene expression associated with toxicity or allergenicity. PubMed was also searched for any evidence of toxicity or allergenicity of Cas9 or DsRed, or of the donor organisms from which these products were originally derived.

**Results:**

While Cas9 nuclease activity can be toxic to some cell types in vitro and hCas9 was found to share homology with the prokaryotic toxin VapC, there was no evidence from previous studies of a risk of toxicity to humans and other animals from hCas9. Although hCas9 did contain an 8-mer epitope found in the latex allergen Hev b 9, the full amino acid sequence of hCas9 was not homologous to any known allergens. Combined with a lack of evidence in the literature of Cas9 allergenicity, this indicated negligible risk to humans of allergenicity from hCas9. No matches were found between the gRNA and microRNAs from either *Anopheles* or humans. Moreover, potential exposure to *dsxF*^*CRISPRh*^ transgenic proteins from environmental releases was assessed as negligible.

**Conclusions:**

Bioinformatic and literature assessments found no convincing evidence to suggest that transgenic products expressed from *dsxF*^*CRISPRh*^ were allergens or toxins, indicating that environmental releases of this population suppression gene drive for malaria vector control should not result in any increased allergenicity or toxicity in humans or animals. These results should also inform evaluations of other GMMs being developed for vector control and in vivo clinical applications of CRISPR-Cas9.

**Supplementary Information:**

The online version contains supplementary material available at 10.1186/s12936-023-04665-5.

## Background

The World Health Organization (WHO) has estimated that in 2020 there were 241 million cases of malaria worldwide, resulting in 627,000 deaths, with countries from Africa shouldering circa 95% of this burden [1]. In recent years, the progress that had previously been observed over the last decade in reducing malaria transmission has stalled, principally due to the emergence of resistance to both insecticide-based vector control against *Anopheles* mosquitoes and pharmaceuticals targeting *Plasmodium* pathogens [1]. This has created the need for additional, complementary approaches to tackle malaria transmission, including novel vector control tools such as engineered gene drives [2, 3].

Typically, engineered gene drives are based on the CRISPR-Cas9 genome editing system and effectuated by a process known as homing [4–6]. The engineered gene drive involves a transgene encoding both the CRISPR-Cas9 endonuclease and guide RNAs (gRNAs) targeting a specific genomic location. The transgene is introduced via germline transformation into its specific genomic target location on one of a pair of homologous chromosomes. Next, in germline cells the Cas9/gRNA ribonucleoprotein (RNP) complex produces a double-stranded break specifically in the same genomic target location of the non-transgenic homologous chromosome. Via homology directed repair (HDR), the transgene and its flanking sequences are pasted into the genomic target location of the homologous chromosome so that both of the homologous chromosomes in that germline cell now contain the transgene. Thus, homing increases the proportion of parental germ cells that are transgenic to above the circa 50% associated with Mendelian inheritance, leading to a sustained increase in the frequency, and, ultimately potential fixation, of the engineered gene drive in the population. This property can be used to propagate transgenes into populations of mosquito vectors of malaria, which can either be used to disrupt reproductive capacity, in the case of population suppression gene drive, or reduce vector competence for *Plasmodium* transmission, in the case of population replacement gene drive [6].

The population suppression gene drive *dsxF*^*CRISPRh*^ is currently being investigated as a potential tool for vector control against malaria [7]. *dsxF*^*CRISPRh*^ encodes (i) a human codon-optimized version of CRISPR-Cas9 endonuclease (hCas9) derived from Cas9 of *Streptococcus pyogenes* (SpCas9), whose expression is controlled by a germline promoter and (ii) the T1 gRNA, which is expressed ubiquitously and acts in concert with hCas9 to target the intron 4-exon 5 boundary of the *doublesex* gene, leading to disruption of the sex-specific transcript *dsxF,* and thus sterility, in females; along with (iii) the DsRed fluorescent marker that was originally sourced from *Discosoma* species and which is expressed in the nervous and ocular systems to facilitate visual detection of the transgene in transgenics [7].

Mosquito vectors of malaria containing such engineered gene drives are considered to be genetically-modified organisms (GMOs), or more specifically genetically-modified mosquitoes (GMMs) [8, 9]. Before any GMM or GMO can be proposed for release in the environment, a rigorous environmental risk assessment (ERA) must be performed [8–11]. As part of this ERA, any potential impacts on human health must be examined, including any potential allergenic or toxic effects from the transgenic proteins [8, 9]. In cases where the GMO is a food crop, allergenicity and toxicity assessments are based on the food to be consumed from that GMO [12, 13]. These evaluations are performed on a case-by-case basis, depending on the nature of the genetic modification, and are based on a weight of evidence approach, as no single investigation can definitively determine allergenicity and toxicity. Initial assessments of the transgenic proteins are tiered to consider amino acid sequence homology comparisons with known allergens or toxins, their sensitivity to pepsin digestion, and evaluation of any new substances or metabolites that they may produce. For example, where a transgenic protein shows less than 35% identity within a segment less than 80 amino acids with a known allergen, it is considered neither to be a known allergen nor likely to be cross-reactive to known allergens [12, 13]. Where a transgenic protein shows greater than 35% identity in a segment of 80 or more amino acids with known allergens, then it should be assessed using serum from individuals sensitized to the identified allergenic source. In contrast to this “35%/80aa cutoff” in allergenicity bioinformatic assessments, there are no specified criteria for homology searches between transgenic proteins and toxins [12–14]. Instead, a weight of evidence approach applied to a tiered evaluation of toxicity has been proposed based on (i) mode of action (ii) amino acid sequence similarity of the transgenic protein to a known toxin, (iii) history of safe use of the protein or source organism, and (iii) probability and level of exposure [12–14]. Alignments of primary amino acid sequences of known toxins with transgenic proteins therefore form an integral part of initial assessment of their potential for toxicity [15]. This tiered approach of substantial equivalence in toxicity assessment has been suggested by the WHO, FAO and OECD [16].

Previously, the first stage of an ERA, problem formulation, was conducted to identify potential harms to protection goals for the environment, as well as human and animal health, from simulated releases of the *dsxF*^*CRISPRh*^ population suppression gene drive in *Anopheles gambiae *sensu lato (*s*.*l*.) in West Africa [17]. That analysis identified 46 plausible pathways to potential harm, nine of which could be evaluated by testing the risk hypothesis that *dsxF*^*CRISPRh*^ transgenic products would not cause increased allergenicity or toxicity. In the present study, amino acid sequences of DsRed and hCas9 were interrogated against those of known toxins or allergens from four different bioinformatic databases. The sequence of the gRNA expressed in *dsxF*^*CRISPRh*^ was also compared with those of *Anopheles* and human microRNAs to examine the potential for disruption to host gene expression that could be associated with increased allergenicity, toxicity or pathogenicity. Finally, literature was examined for any evidence of toxicity or allergenicity of the transgenic proteins themselves, or of the donor organisms from which they were originally derived.

## Methods

### Bioinformatic analyses

#### Search strategies

Amino acid sequences of the transgenic proteins DsRed and hCas9, and nucleotide sequences of the T1 gRNA, all of which are expressed from the engineered gene drive *dsxF*^*CRISPRh*^, are shown in Table 1. Sequence searches were conducted between 5 and 15th November 2022 and exploited four bioinformatic repositories:

- NCBI, the National Centre for Biotechnology Information of the National Library of Medicine of the National Institutes of Health of the USA (https://www.ncbi.nlm.nih.gov/). The 30^th^ October 2022 update of Expressed Sequence Tags (EST) databases of NCBI contained 77,393,133 cDNA sequences. The 5th November 2022 update of the Transcript Reference Sequences (TRS) databases of the NCBI contained 39,247,328 cDNA sequences.
- UniProt; maintained by the European Molecular Biology Laboratory’s European Bioinformatics Institute (EMBL-EBI), Swiss Institute of Bioinformatics (SIB) and Protein Information Resource at Georgetown University, USA (https://www.uniprot.org/). The UniProtKB database is a comprehensive, high-quality and freely accessible protein database made up of both the Swiss-Prot protein database that, as of 12th October 2022, contained 568,363 manually annotated and reviewed entries and the TrEMBL translated nucleotide database containing 229,928,140 automatically annotated entries.
- COMPARE database: Comprehensive Protein Allergen Resource (COMPARE) database, 2022 update (http://www.comparedatabase.org/), consists of a repository of 2463 peer-reviewed protein sequences of known or putative allergens, and is maintained as a collaborative effort of the Health and Environmental Sciences Institute and Protein Allergenicity Technical Committee with programmatic support from the Joint Institute for Food Safety and Nutrition at the University of Maryland.
- AllergenOnline database maintained by the Food Allergy Research and Resource Program of the University of Nebraska: version 21 (http://www.allergenonline.org/) with 2,233 allergen sequences, updated on 14th February 2021.

**Table 1:** Amino acid or nucleotide sequences of transgenic products expressed in *dsx*^*CRISPRh*^ and amino acid sequence of the allergen Hev b 9

Product	Source	Amino acid or nucleotide sequence
**DsRed** **(226 aa)**	Coral *Discosoma *sp.	MVRSSKNVIKEFMRFKVRMEGTVNGHEFEIEGEGEGRPYEGHNTVKLKVTKGGPLPFAWDILSPQFQYGSKVYVKHPADIPDYKKLSFPEGFKWERVMNFEDGGVVTVTQDSSLQDGCFIYKVKFIGVNFPSDGPVMQKKTMGWEASTERLYPRDGVLKGEIHKALKLKDGGHYLVEFKSIYMAKKPVQLPGYYYVDSKLDITSHNEDYTIVEQYERTEGRHHLFL
**hCas9** **(1423 aa)**	*Streptococcus pyogenes*	MDYKDHDGDYKDHDIDYKDDDDKMAPKKKRKVGIHGVPAADKKYSIGLDIGTNSVGWAVITDEYKVPSKKFKVLGNTDRHSIKKNLIGALLFDSGETAEATRLKRTARRRYTRRKNRICYLQEIFSNEMAKVDDSFFHRLEESFLVEEDKKHERHPIFGNIVDEVAYHEKYPTIYHLRKKLVDSTDKADLRLIYLALAHMIKFRGHFLIEGDLNPDNSDVDKLFIQLVQTYNQLFEENPINASGVDAKAILSARLSKSRRLENLIAQLPGEKKNGLFGNLIALSLGLTPNFKSNFDLAEDAKLQLSKDTYDDDLDNLLAQIGDQYADLFLAAKNLSDAILLSDILRVNTEITKAPLSASMIKRYDEHHQDLTLLKALVRQQLPEKYKEIFFDQSKNGYAGYIDGGASQEEFYKFIKPILEKMDGTEELLVKLNREDLLRKQRTFDNGSIPHQIHLGELHAILRRQEDFYPFLKDNREKIEKILTFRIPYYVGPLARGNSRFAWMTRKSEETITPWNFEEVVDKGASAQSFIERMTNFDKNLPNEKVLPKHSLLYEYFTVYNELTKVKYVTEGMRKPAFLSGEQKKAIVDLLFKTNRKVTVKQLKEDYFKKIECFDSVEISGVEDRFNASLGTYHDLLKIIKDKDFLDNEENEDILEDIVLTLTLFEDREMIEERLKTYAHLFDDKVMKQLKRRRYTGWGRLSRKLINGIRDKQSGKTILDFLKSDGFANRNFMQLIHDDSLTFKEDIQKAQVSGQGDSLH***EHIANLAG***SPAIKKGILQTVKVVDELVKVMGRHKPENIVIEMARENQTTQKGQKNSRERMKRIEEGIKELGSQILKEHPVENTQLQNEKLYLYYLQNGRDMYVDQELDINRLSDYDVDHIVPQSFLKDDSIDNKVLTRSDKNRGKSDNVPSEEVVKKMKNYWRQLLNAKLITQRKFDNLTKAERGGLSELDKAGFIKRQLVETRQITKHVAQILDSRMNTKYDENDKLIREVKVITLKSKLVSDFRKDFQFYKVREINNYHHAHDAYLNAVVGTALIKKYPKLESEFVYGDYKVYDVRKMIAKSEQEIGKATAKYFFYSNIMNFFKTEITLANGEIRKRPLIETNGETGEIVWDKGRDFATVRKVLSMPQVNIVKKTEVQTGGFSKESILPKRNSDKLIARKKDWDPKKYGGFDSPTVAYSVLVVAKVEKGKSKKLKSVKELLGITIMERSSFEKNPIDFLEAKGYKEVKKDLIIKLPKYSLFELENGRKRMLASAGELQKGNELALPSKYVNFLYLASHYEKLKGSPEDNEQKQLFVEQHKHYLDEIIEQISEFSKRVILADANLDKVLSAYNKHRDKPIREQAENIIHLFTLTNLGAPAAFKYFDTTIDRKRYTSTKEVLDATLIHQSITGLYETRIDLSQLGGDKRPAATKKAGQAKKKK
**Hev b 9** **enolase, isoform 1** **(445 aa) (**Accession number: CAC00532)	*Hevea brasiliensis*	MAITIVSVRARQIFDSRGNPTVEADVKLSDGYLARAAVPSGASTGIYEALELRDGGSDYLGKGVSKAVENVNIIIGPALVGKDPTDQVGIDNFMVQQLDGTVNEWGWCKQKLGANAILAVSLAVCKAGAHVKGIPLY***EHIANLAG***NKNLVLPVPAFNVINGGSHAGNKLAMQEFMILPVGASSFKEAMKMGAEVYHHLKSVIKKKYGQDATNVGDEGGFAPNIQENKEGLELLKTAIAKAGYTGKVVIGMDVAASEFYGSDQTYDLNFKEENNNGSQKISGEALKDLYKSFVAEYPIVSIEDPFDQDDWAHYAKLTSEIGEKVQIVGDDLLVTNPKRVEKAIKEKACNALLLKVNQIGSVTESIEAVKMSKRAGWGVMASHRSGETEDTFIADLSVGLATGQIKTGAPCRSERLAKYNQLLRIEEELGSEAVYAGANFRKPVEPY
**T1 gRNA** **(96 bases)**	*Anopheles gambiae;* synthetic	GTTTAACACAGGTCAAGCGGGTTTTAGAGCTAGAAATAGCAAGTTAAAATAAGGCTAGTCCGTTATCAACTTGAAAAAGTGGCACCGAGTCGGTGC

Potential epitope in hCas9 identified in 8-mer sliding window search reveals sequence identity to Hev b 9 and is highlighted in bold italics. The genomic target sequence of T1 gRNA is underlined

### hCas9 or DsRed homology searches

Using TBLASTN bioinformatics program version 2.13.0 + , the amino acid sequence of hCas9 or DsRed transgenic protein was used to search NCBI TRS and EST databases. Searches were limited by Entrez query “toxin” or “allergen”. Only search returns with Expected values (“E values”) of 10 or below were returned. Algorithm parameters were based on program default settings of Matrix—BLOSUM 62, Gap Costs—Existence: 11 Extension: 2; Compositional adjustments—Conditional compositional score matrix adjustment. Any resultant protein sequence alignments were saved on the NCBI server.

UniProtKB version 2022_04 was searched using the keyword “toxin” to identify 1,280,940 entries, which were downloaded from the UniProt website as a compressed FASTA file (“UniProt-Toxin”). Separately, the UniProtKB database was searched using the keyword “allergen” to identify 53,290 entries, which were downloaded from the UniProt website as compressed FASTA file (“UniProt-Allergen”). Using standalone BLAST + software for Windows [18], UniProt-Toxin and UniProt-Allergen were converted to BLAST protein databases. Using BLASTP 2.13.0 + , sequence alignments with the full-length amino acid sequences of both Cas9 and DsRed were performed on the amino acid sequences from both the BLAST UniProt-Toxin and BLAST UniProt-Allergen protein databases.

For both NCBI and UniProt searches, alignments for allergens were considered significant where the protein contained a stretch of 80 or greater amino acids that showed 35% or more identity [12]. Any alignments which were returned with an E value less than 10 from bioinformatic toxicity assessments were examined further manually for significant sequence identity over a substantive length of protein. Considerations for significance during these manual examinations were informed by a number of observations from the literature. Firstly, in comparative modelling of proteins, primary amino acid sequences are used to infer three-dimensional protein secondary structures, typically yielding high levels of accuracy above 50% sequence identity but highly inconsistent outcomes when sequence identity fails below 30% [19–21]. As articulated by Negi et al. [22], sequences with 35% identity are generally considered to have similar three-dimensional structures [23–25]. Indeed, their systematic analysis of over 10,000 manually curated toxin sequences led to the proposal that 35% sequence identity level could be used for functional grouping of toxins [22]. Secondly, the 80aa cut-off for allergenicity testing adopted by Codex Alimentarius was reportedly based on the length of stretches of conserved amino acids corresponding to function domains in proteins [26]. This assertion is consistent with studies of the free energy of unfolding of protein domain structures [27], or the distribution of sizes of protein domain boundaries from a three-dimensional database of 1882 sequence-dissimilar protein domains [28], which indicate a narrow range of around 100 amino acids for optimal sizes of protein domains. Adoption of an 80 amino acid as a cut-off for domain sizes of toxins would thus represent a conservative estimate. Therefore, for evaluation of alignments between transgenic proteins and toxins, alignments with 35% or more amino acid identity over a segment of 80 or more amino acids were considered to be significant.

The COMPASS (COMPare Analysis of Sequences with Software) program available from the COMPARE allergen database was used to search for stretches of 35% or greater identity over 80 or greater amino acids with DsRed and hCas9. Algorithm input parameters were based on program default settings of E value 10, Word Size 2, Gap Open and Gap Extension Penalties 10/2. On the recommendation of Codex Alimentarius [12], COMPARE was further searched by 80 amino acid segments of transgenic protein segments in order to identify structural motifs, much shorter than the intact protein, which might contain a conformational IgE binding epitope or could help to identify potentially cross-reactive proteins that are not true homologues of an allergen that have significant local identities that might provide an immunological target for IgE antibodies in those with allergies to the matched allergen. A match of 35% or more over 80 or greater amino acids with a known allergen suggested further testing for possible cross-reactivity. In addition, based on hypothetical epitope sizes, windows of 8 amino acids of the transgenic query sequence were also compared with known allergens in the COMPARE database.

Amino acid sequences of transgenic proteins DsRed and hCas9 were also used to search the AllergenOnline database using FASTA36 with scoring matrix BLOSUM 50 to identify potential allergenic proteins. Search returns were limited to E value of 10 or below. A match of 35% or more over 80 or greater amino acids with a known allergen suggested further testing for possible cross-reactivity. AllergenOnline was further searched by 80 amino acid segments of transgenic protein segments in order to identify structural motifs, much shorter than the intact protein, which might contain a conformational IgE binding epitope. A match of 35% or more over 80 or more amino acids with a known allergen indicated further testing for possible cross-reactivity. In addition, windows of 8 contiguous amino acids of the transgenic query sequence were also compared with known allergens in the AllergenOnline database.

### gRNA nucleotide search strategy

The nucleotide sequence of the T1 gRNA is shown in Table 1. This is expected to be constitutively and ubiquitously expressed from the *U6* promoter in *dsxF*^*CRISPRh*^ [7] and thus was assumed to be present in the saliva of *dsxF*^*CRISPRh*^ GMMs. The nucleotide sequence of the gRNA was examined for similarities to known microRNAs to establish whether they might exert allergenic or toxic effects via the potential to alter endogenous gene expression in either GMMs or humans that might be bitten by the GMMs.

The miRBase is the primary public repository and online searchable database for microRNA (miRNA) sequences and annotation, containing 38,589 entries (http://www.mirbase.org/). It was established in the UK by the Wellcome Sanger Institute for genome research and is hosted by the University of Manchester. Searches using both gRNA nucleotide sequences from *dsxF*^*CRISPRh*^ were conducted on the miRBase22.1 data release from October 2018 using the BLASTN search method to identify any similar mature miRNA sequences from all organisms in the database, including *An*.* gambiae* and *Homo sapiens*. Search results were considered significant where E values were less than 0.01 [29].

### Literature review

#### Search strategy

The PubMed biomedical literature database (https://pubmed.ncbi.nlm.nih.gov/), was used as the primary data source for scientific literature on allergy and toxicity. The main objective of the searches was to identify evidence of toxicity or allergenicity of (i) ‘the transgenic proteins’ hCas9 and DsRed; and (ii) ‘the source organisms’ of the transgenic proteins, *Streptococcus pyogenes* and *Discosoma* spp., respectively. Combinations of different search strings were used, involving the terms ‘allergen’ or ‘allergenic’, ‘toxin’ or ‘toxicity’, ‘DsRed’; ‘Cas9’; ‘*Discosoma’* or *‘Streptococcus pyogenes*’. Each search string was conducted to obtain results from inception of search engine until the 31st October 2022. Results were downloaded to the Endnote reference manager software (Endnote V. X9.2) for storage and review. Abstracts of identified publications were examined against inclusion and exclusion criteria. The full text of surviving articles was subsequently examined against selection criteria. Based on bioinformatic and initial literature results, further specific literature searches were used to identify additional literature relevant to specific putative toxins or allergens of interest.

#### Eligibility

### Selection criteria

Research articles and published laboratory studies were included for review when they:

- Were structured with introduction, materials and methods, results, and discussion sections or equivalent
- Were authored in English
- Were published between inception of the PubMed search engine until date of completions of searches in this study, 31st October 2022
- Provided in vitro*, *in vivo or ex vivo evidence of acute or chronic toxicity or allergenicity (or lack thereof) caused directly by (a) Cas9; (b) DsRed; (c) any of the components or breakdown products of (a) and (b), or (d) *Discosoma* spp., or *S*.* pyogenes*
- Were pathological studies involving source organism/s which established a mechanistic cause of disease effects
- Provided evidence of allergenicity through the route of IgE antibody elicitation
- Were available as full-text items through either open access, or through Imperial College London library and online literature facilities.

### Defining toxicity and allergenicity

The WHO has defined a “toxic agent” as “anything that can produce an adverse biological effect” and “toxicity” as “the capacity of a substance to cause injury to a living organism. A highly toxic substance will cause damage in small quantities, while a substance of low toxicity will need large quantities to produce an effect. Toxicity is also dependent on the portal of entry, the time frame of exposure and the latent period” [30]. With reference to genetically modified foods, the Society of Toxicology has defined potential toxicity to include “inherent toxicity of the transgenes and their products, and unintended (pleiotropic or mutagenic) effects resulting from the insertion of new genetic material into the host genome” [31]. Therefore, for this analysis, toxicity was assumed in any study that demonstrated harm or impairment to function of a cell, or component of an organism, or cause cell death in cells or an organism, including via mutagenic effects.

Allergenicity is the potential of a substance to cause an allergy and can be caused by a variety of immunological mechanisms, however IgE-mediated allergy represents the main form of allergy, that causes the most severe reactions and the only form causing life-threatening reactions [32]. An allergen, in the context of an IgE food allergy, exerts its effects in two stages: first, (i) sensitisation where no symptoms occur while the capacity of the immune system to react increases dramatically, and later (ii) elicitation (provocation) with clinical manifestations [32]. This form of IgE-mediated allergy has been the focus of risk assessments of allergenicity of GMOs and novel foods [12, 32–37].

An allergen is further defined by AllergenOnline as a protein that has been demonstrated to “specifically bind IgE using sera with individuals with clear allergies to the source of the gene/protein and further that the protein causes basophil activation or histamine release, skin test reactivity or challenge test reactivity using subjects allergic to the source.” Therefore, for this analysis, allergenicity was defined as any substance that has evidence of eliciting an IgE response, in vivo or in vitro*,* both to the protein source or to protein itself.

## Results

### hCas9 toxicity

#### Bioinformatic assessment of hCas9 toxicity

NCBI TRS and EST database searches yielded four entries with an E-value below 10, but none had 35% or greater identity over 80 or more amino acids to known or putative toxins. Searches of BLAST UniProt-Toxin protein databases with hCas9 identified six proteins with E-values below 10 (see Table 2). Only one of these sequence alignments was significant: entry H1D476 encoding a Type II toxin-antitoxin system VapC family toxin from *Fusobacterium necrophorum* subsp. funduliforme, which showed 43% identity to hCas9 over an alignment length of 119 amino acids (shown in blue in Table 2).

**Table 2:** Bioinformatic search results for sequence identity with hCas9

Database	Program	Keyword limitation	Accession #	Organism	Description	Length (aa)	*E* value	% identity	Alignment length (aa)
NCBI-TRS	TBLASTN	Toxin	XM_040336796.1	*Rana temporaria*	Multidrug and toxin extrusion protein 1-like (LOC120926671), transcript variant X3, mRNA	1616	0.87	38	47
NCBI-TRS	TBLASTN	Toxin	XM_040336794.1	*Rana temporaria*	Multidrug and toxin extrusion protein 1-like (LOC120926671), transcript variant X1, mRNA	1740	0.89	38	47
NCBI-TRS	TBLASTN	Toxin	XM_040336795.1	*Rana temporaria*	Multidrug and toxin extrusion protein 1-like (LOC120926671), transcript variant X2, mRNA	1742	2.0	36	47
NCBI-EST	TBLASTN	Toxin	CMD69_G06_40	*Sus scrofa*	MNMPM3 Sus scrofa cDNA clone PPSUBLIB_56C06 5', mRNA sequence	594	10.0	42	38
BLAST UniProt-toxin	BLASTP	Toxin	A0A502HM27	*Brevibacillus laterosporus (Bacillus laterosporus)*	CRISPR-associated endonuclease Cas9	1092	2e−25	26	549
BLAST UniProt-toxin	BLASTP	Toxin	H1D476	*Fusobacterium necrophorum *subsp.* funduliforme*	Type II toxin-antitoxin system VapC family toxin	117	2e−18	43	119
BLAST UniProt-toxin	BLASTP	Toxin	A0A1G4RN64	*Lachnospiraceae bacterium*	Nucleotidyl transferase AbiEii/AbiGii toxin family protein	249	1.8	27	103
BLAST UniProt-toxin	BLASTP	Toxin	A0A1H2T7K0	*Lachnospiraceae bacterium*	Nucleotidyl transferase AbiEii/AbiGii toxin family protein	249	3.9	27	103
BLAST UniProt-toxin	BLASTP	Toxin	A0A2H0VRT0	*Chlamydiae bacterium*	Nucleotidyl transferase AbiEii/AbiGii toxin family protein	253	4.5	25	119
BLAST UniProt-toxin	BLASTP	Toxin	A0A7X8NY49	*Citrobacter freundii*	Insulinase family protein	497	0.47	22	168
BLAST UniProt-allergen	BLASTP	Allergen	A0A834R755	*Sarcoptes scabiei*	Mite allergen Der p 3	595	3.1	30	56
BLAST UniProt-allergen	BLASTP	Allergen	A0A3S2L044	*Chilo suppressalis*	Uncharacterized protein	194	6.9	45	31

**Fig. 1:**
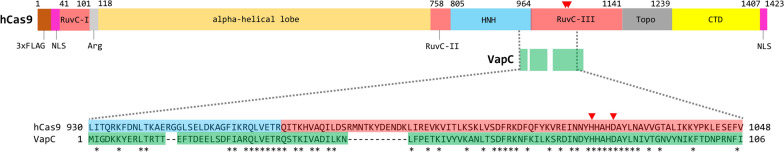
Sequence alignment between hCas9 and VapC. The domains within the hCas9 protein are illustrated in colour in the top box graphic [43], with all numbers corresponding to amino acids in hCas9, and regions of alignment to VapC (green box graphic) indicated by the dotted lines. From N- to C-terminus of hCas9, domains are “3 × FLAG” (coloured brown): FLAG epitopes in three tandem repeats positioned at the N-terminus of hCas9; “NLS” (purple): Nuclear localization signal; “RuvC-I” (red): RuvC domain I; “Arg” (silver): Arginine-rich domain; “Alpha-helical lobe” (orange); “RuvC-II” (red): RuvC domain II; “HNH” (blue): NHN nuclease domain; “RuvC-III” (red): RuvC domain III; “Topo” (dark grey): topo-homology domain; “CTD” (yellow): C-terminal domain [43]. Red triangles indicate catalytic residues in the RuvC-III domain [43]. Amino acid sequence alignments between hCas9 and VapC are shown below box graphics, with blue- and red- coloured amino acids from NHN and RuvC-III domains of hCas9, respectively, and VapC amino acids shown in green below [43]. Identical amino acids between both proteins are indicated by asterisks

In terms of mode of action, the VapC toxin encodes a sequence specific endoribonuclease that cleaves RNA species, such as the initiator tRNA^fMet^ or 23S rRNA, which are specifically components of bacterial mRNA translation [38–41]. In the hCas9 endonuclease, the HNH domain cleaves the DNA strand that is complementary to the gRNA target sequence, while the RuvC domain cleaves the non-target DNA strand, so that both domains must act in concert to produce a double-stranded break (DSB) in a specific DNA sequence. In *F*.* necrophorum*, the *VapC* gene is predicted to encode a protein of 117 amino acids [42], 33 and 73 of which align within the HNH and RuvC-III domains found between amino acids 805-964 and 965-1141 of hCas9, respectively [43] (see Fig. 1). While this alignment contains neither of the amino acids serving as catalytic residues in the HNH domain, the HHAHDAYN motif that is common to both the VapC protein and the RuvC-III domain of hCas9 contains two of the four catalytic residues of the RuvC active site (Fig. 1) and is a highly conserved motif in Cas9 homologues from multiple species of bacteria [43]. Amino acid sequence alignment between VapC and hCas9 is thus based on a similar nucleic acid cleavage capability between both proteins.

In terms of history of safe use of the protein or source organism, when the UniProt database was searched with the predicted amino acid sequence of this VapC protein, 341 hits from a range of prokaryotic species were identified with an E-value below 10 (see Additional file 1: File S1). Of these entries, 239 (70%) included the term “Cas9”. Thus, VapC shows sequence identity to a wide range of Cas9 homologues in heterogenous bacterial species that are widely distributed throughout nature [44]. Similarly, humans and other animals are regularly exposed to *F*.* necrophorum*, as it is commonly found within the alimentary tract; in studies based in London accounted for 10% and 21% of acute and recurrent sore throats, respectively [45–48]. However, the action of the VapC toxin does not itself directly cause symptoms in patients; rather bacterial toxin-antitoxin systems such as VapC consist of both a protein toxin that can interfere in essential bacterial physiological processes to inhibit cell growth and an antitoxin to counteract the bactericidal or bacteriostatic activity of the toxin [49]. There are three broad functions ascribed to such toxin-antitoxin systems, each of which may confer a survival advantage to their bacterial hosts [49]. Firstly, in “post-segregational killing” a plasmid encoding both the toxin and a less stable antitoxin leads to the death of plasmid-free offspring that are incapable of producing antitoxin [50]. Secondly, in “abortive infection” bacteriophage multiplication in a population of bacteria is inhibited by activation of the toxin-antitoxin system when cells infected with bacteriophage undergo “altruistic” cell death [51, 52]. Thirdly, in “persister formation” a subset of bacterial cells in a population develops tolerance to antibiotics or other environmental stressors as a result of the toxin inhibiting biochemical functions normally targeted by those stressors, leading to dormancy rather than death in those cells [53]. Therefore, any toxicity of VapC is restricted to prokaryotes via its inhibitory effects on mRNA translation, and thus cell growth, specifically in bacteria.

### Literature assessment of hCas9 toxicity

Biting is a likely route of exposure to material from *dsxF*^*CRISPRh*^ mosquitoes while they are seeking bloodmeals from human or animal hosts. However, *dsxF*^*CRISPRh*^ Cas9 is under the control of the *zero population growth* (*zpg*) promoter which drives expression in the germline of both males and females from late pupal developmental stages. Cas9 is therefore not expected to be expressed in the saliva of *dsxF*^*CRISPRh*^ transgenics [7]. In the case of accidental, incidental ingestion of *dsxF*^*CRISPRh*^ mosquitoes by humans or animals, Cas9 would also be subject to pepsin digestion [54] so that exposure via this route is expected to be negligible. In general, the levels of Cas9 transgenic protein in any given individual *dsxF*^*CRISPRh*^ mosquito would be insignificant in terms of biomass within the environment [55].

Nonetheless, Cas9 is a nuclease capable of inducing DSBs in DNA and, therefore, has the potential to be mutagenic. Recognition of its genomic target sequence is facilitated via the particular sequence of its gRNA so that its endonuclease activity can be designed to be highly target-specific [56]. By altering the circa 20 nucleosides target recognition sequence of the gRNA, Cas9 can be directed towards specific nuclease activity against almost any DNA target of interest. While Cas9-induced DSBs other than at the intended target sequence, known as “off-target effects”, have been observed in some studies [57, 58], many other i*n vivo* studies have shown low (< 1%) or no off-target effects through therapeutic proof-of-concept use of Cas9 [59–62]. Cas9 has been used to investigate carcinogenic factors in vivo [63] as well as other clinical conditions with no detectable off-target effects [64–68]. In some in vitro studies, however, indels ranging from small to large, as well as chromosomal translocations, chromothripsis and chromosomal aberrations, have been observed [69–76]. For example, Kosicki et al. detected “on-target mutagenesis”, such as large deletions and insertions, at target sites in mouse and human cell lines [70]. To understand the origins of these deletions, the authors generated a library of mouse embryonic stem cells deficient in 32 DNA repair genes based on a single clone constitutively expressing Cas9, and found that the frequency of large deletions increased when genes essential for non-homologous end-joining (NHEJ) were impaired, and decreased when genes required for microhomology-mediated end joining (MMEJ) were disrupted [77]. Depending on the specific cell type or stage of cell cycle, some DNA repair mechanisms, whether HDR, NHEJ or MMEJ, may be favoured over others, which thus may determine the propensity for, and characteristics of, any such on-target effects [78–80]. For example, prolonged in vitro intracellular expression of Cas9 in human pluripotent stem cells caused DSBs in DNA that were toxic via P53 inhibition of HDR, a mechanism which appeared to be cell-specific and related to the early developmental status of the cell types [81, 82]. Kosicki et al. urged caution when using high concentrations of nucleases and proposed further investigations using inducible Cas9 or gRNAs of different target site binding affinities [77]. Indeed, judicious choice of target sites and design of gRNAs has been widely demonstrated to improve the target specificity of Cas9 in a wide range of systems [57–68, 83–96], including in in vivo human studies [97].

As can be the case for ectopic expression of other heterologous proteins, constitutive intracellular expression of Cas9 can also be toxic due to stresses on protein homeostasis. For example, constitutive intracellular expression, but not transient expression, of Cas9 is toxic in the single-cell alga *Chlamydomonas reinhardtii* [98], rice blast fungus *Magnaporthe oryzae* [99] and the protozoan parasite *Toxoplasma gondii* [100]. Ferreira et al. [101] found that strong in vivo expression of Cas9 in zebrafish muscle fibres induced cell toxicity but attributed this to disruptions to cellular protein homeostasis leading to apoptosis. The method of delivery used for Cas9, rather than Cas9 activity by itself, may also cause toxicity. In a study by Li et al. [82], two different delivery methods for Cas9 were compared under the same experimental settings. Authors found that an adenovirus vector in vitro resulted in attenuated toxicity seen in CD34 + haematopoietic stem cells when compared with the electroporation method of delivery. Similar results were found in vivo in mice muscle myofiber degeneration and repair, when adenovirus vector showed no significant muscle cell damage, compared with electroporation methods [102]. Chromatin environment of the targeted locus has also been found to affect DSBs associated toxicity as a result of CRISPR/Cas9 editing [103], though this has been disputed by Friskes et al. who attribute major determinants of toxicity to be related to cutting efficiency and off-target DSBs, as opposed to chromatin features [104]. Holmgaard et al. [105] created mutations in the *vascular endothelial growth factor A* (*VEGFA*) gene in retinal pigment epithelium in mice by performing subretinal injections of lipoplexes containing a range of concentrations of guide RNAs complexed with Cas9 as RNPs. Toxicity was only induced at high, but not lower, concentrations of RNP. Therefore, the authors concluded that RNP-based delivery in vivo was a potential strategy for treatment of retinal diseases in humans. Consistent with these observations, Garrood et al. [106] reported that Cas9 off-target mutagenic effects could not be detected in the genomes of *dsxF*^*CRISPRh*^ GMMs, despite such events being observed in other gene drive strains that expressed hCas9 from the *vasa2* promoter and gRNAs targeting disparate genomic targets. The specificity of hCas9 DNA endonuclease activity in *dsxF*^*CRISPRh*^ GMMs appears to be a consequence of both the choice of sequence of the T1 gRNA specifying the genomic target and more restricted spatiotemporal expression of hCas9 from the *zpg* promoter, compared to *vasa2*. Lower levels of hCas9 expression from the *zpg* promoter thus appeared to favour the absence of detectable off-target nuclease activity. Overall, therefore, any toxicity of Cas9 is associated with high cellular levels of its expression or concentration.

There are numerous animal studies where Cas9 has been expressed in vivo, without any toxic effects observed. Qiu et al. [68] showed expression of Cas9 in vivo in mice resulted in no off-target mutagenesis or evidence of toxicity. Reisman et al. [107] used Cas9 in vivo in a murine model to successfully suppress leukaemia progression, with minimal toxicity observed. Xiong et al. [108] showed that nanoparticle delivery of Cas9 resulted in low toxicity and high biological safety both in vitro and in vivo in mice. Han et al. [109] demonstrated safe and effective use of Cas9 in vivo, in murine models for haemophilia therapy. Wei et al. [110] showed effective therapeutic in vivo transplantation of Cas9 gene-corrected autologous hepatocytes into mice with Wilson’s disease with no toxicity seen. Many other in vivo studies involving expression of Cas9 demonstrated no, negligible or low toxic effects [94, 111–118].

Human exposure to Cas9 can take the form of either ex vivo or in vivo therapeutic applications [61, 76, 119, 120]. In ex vivo therapies, autologous cells are removed from the patient and modified in vitro with a vector encoding Cas9 or using recombinant RNPs. Once the genomic editing has been performed, the edited cells are infused back into the patient. Any potential toxicity of Cas9 from ex vivo therapeutic applications would likely arise from *indirect effects*, such as the introduction of off- or on-target mutations in, or toxic effects on, the edited cells [121]. In the case of in vivo therapeutic applications, Cas9 is introduced systemically into the patient via viral or lipid nanoparticle gene delivery systems. In vivo therapies could thus expose patients to *direct effects* of potential toxicity from Cas9 [121].

For ex vivo therapies, Cas9 has been exploited as a gene editing tool for cancer treatment, with minimal toxicity observed [72, 122]. For example, Foy et al. [123] reported the ex vivo non-viral use of CRISPR-Cas9 to treat 16 patients with refractory solid tumours. In T cells obtained from patients, two T cell receptor genes were knocked out and replaced with mutant neoantigen-specific T cell receptors that been previously identified from circulating T cells in patients. After chemotherapy to deplete pre-existing lymphocytes, the modified autologous T cells were infused into patients. There were only two adverse events associated with the T cell therapy, one involving mild cytokine release and neutropenia and another involving grade 3 encephalitis which was resolved with corticosteroid treatment, demonstrating the safety of this approach [123, 124]. The immunotherapeutic use of Cas9 to edit T-cells through disruption of the PD-1 gene has also now entered Phase I clinical trials [125, 126]. A further Phase I clinical trial has used CRISPR-Cas9 engineered CAR19 universal T cells to treat children with refractory B cell leukaemia, with all primary safety objectives met, demonstrating feasibility and therapeutic potential of this immunotherapy [127]. Frangoul et al. [128] also reported that two patients, one with transfusion-deficient beta-thalassaemia (TDT) and one with sickle cell disease were administered with a Cas9 to target the BCL11A transcription factor. More than a year later, both patients had high levels of allelic editing with no off-target effects observed. While the first patient exhibited two serious side effects, one was attributed to a case history of viral hepatitis, while the other, pneumonia, resolved within 28 days. The second patient had three serious adverse events, which were all attributed to the administered Cas9 being enriched with CD34 + cells, which may have contributed to the delay in lymphocyte recovery. A further clinical trial in Phase III of development is underway, utilizing ‘Exa-cel’, an investigational, autologous, *ex-vivo* CRISPR/Cas9 gene-edited therapy being evaluated for patients with sickle-cell disease or transfusion-dependent beta-thalassaemia [129]. Having provided robust evidence of potential as a one-time functional cure for these diseases, in previous clinical trials, two patients out of seventy-five reported serious adverse events, both of which were resolved [130].

With respect to in vivo therapeutic applications of Cas9, Gillmore et al. [97] reported delivery of intravenous injections of mRNA encoding Cas9 and a gRNA targeting the transthyretin gene encapsulated in lipid nanoparticles in six patients with life-threatening transthyretin amyloidosis. Safety assessments of those patients during the first 28 days of the clinical trial revealed few adverse events, all of which were mild in grade. A second clinical trial uses CRISPR-Cas9 to disrupt the kallikrein protein to counteract altered levels of the peptide hormone bradykinin in patients with hereditary angioedema, which results in severe swelling of the limbs and abdomen [131]. The intervention involves Cas9 encoding mRNA, along with its gRNA, delivered in lipid nanoparticles and injected directly into the patients’ bloodstream which then travels to, and gets expressed in, the liver [131]. Swelling attacks went from one to three a month in two patients, and from up to seven a month to none in another, so that all three were able to stop taking their anti-swelling pharmaceuticals [131]. A third clinical trial is underway involving adeno-associated virus in vivo delivery of Cas9, for gene editing of photoreceptor cells to treat blindness, with preliminary results revealing a ‘favourable safety profile’ [132], with two patients seeing improvements in their vision [133]. Another ongoing in vivo therapeutic study is directed at the treatment of cervical intraepithelial neoplasia and cervical cancer by directly injecting plasmid DNA encoding Cas9 that targets the human papilloma virus [119]

### hCas9 allergenicity

#### Bioinformatic assessment of hCas9 allergenicity

NCBI TRS and EST database searches yielded no entries with an E-value below 10. Searches of BLAST UniProt-Allergen protein databases with hCas9 identified two proteins with E-values below 10 (see Table 2). However, neither sequence alignment was significant. Using the COMPASS to search the COMPARE database, no entries were identified with E values below 10 (see Table 2). Sliding 80-mer searches identified no positive hits. Searches of the AllergenOnline database also identified no entries with E values below 10 and no sliding 80-mer positive hits.

In searches of both COMPARE and AllergenOnline, of the 1416 stretches of 8 amino acids identified in hCas9, one 8-MER sequence match, ‘EHIANLAG’, was also found in the protein allergen Hev b 9 (enolase isoform 1 from *Hevea brasiliensis*; NCBI accession number CAC005320; highlighted in blue in Table 1.) Epitopes on different proteins, as a rule evolutionarily related, may be identical or similar enough to bind to the same IgE molecules. Cross-reactivity, defined as the sensitization of one allergen causing the immune system to respond to another allergen as a result of shared epitopes [134], could then potentially occur. There are two levels of cross reactivity; (i) limited to IgE-binding without observed adverse effects (cross sensitization) or (ii) IgE-binding which also confers clinical reactivity (cross-allergenicity) [32]. Cross-reactivity on the level of IgE-binding is more common and widespread than clinical cross-reactivity [32]. The in vitro demonstration of cross-reactivity between two allergens in terms of IgE-binding is no proof of clinical cross-reactivity, and only means that one important pre-requisite for clinical reactivity is present [32, 134]. Furthermore, even the presence of IgE to a specific allergen may result in negative clinical challenges, as shown in a study exploring soybean allergenicity [135].

Hev b 9 is one of 15 allergens officially listed by the WHO as being identified in causing Natural Rubber Latex Allergy (NRLA) [136]. Some of these identified proteins such as Hev b 5 and Hev b 6.01, are also found in other “lactiferous” or latex-excreting plants, and are associated with asymptomatic sensitization, as well as IgE-mediated hypersensitivity [136]. Of importance, Hev b 9, whilst sharing one epitope with hCas9, shares 65% sequence identity and 65 epitope matches with Human enolase isoform 1, found in mammalian tissue and functions as a glycolytic enzyme [137]. Human enolase 1 or alpha enolase, is one of three enolase isoenzymes found in mammals; and was found to have significant sequence identity to Hey b 9 but was not found to contain the ‘EHIANLAG’ epitope found in Cas9. Fagerberg et al. 2014 analysed expression of Human enolase 1 in tissue samples from 95 human individuals to determine tissue specificity of various protein-coding genes, and found ubiquitous expression in kidney, oesophagus and 25 other tissues [137].

The identical peptide match method using a peptide length of six amino acids has attracted criticism, since it generates many false positives in testing of potential allergenicity [138–141]. Moreover, even in linear B-cell epitopes some amino acids can be replaced without loss of antibody binding and, as a consequence, Codex Alimentarius does not include the criterion of six identical amino acids in their guidelines [12], but rather proposed instead that the scanning peptide size should be based on a scientifically-justified rationale. European Food Safety Authority (EFSA) guidance on the assessment of the allergenicity of GM foods aligns with the recommendations of the Codex Alimentarius for in silico testing for prediction of potential allergenicity is recommended [142]. However, EFSA recommend that peptide match of complete identity over six contiguous amino acids to known allergens is—on its own—associated with very poor specificity and its relevance is doubtful [32].

Furthermore, AllergenOnline states that searches can be performed where it was not possible to find examples of an isolated identity match of six or eight amino acids with cross-reactive proteins unless there was at least a 35% identity match over 80 amino acids. In further consideration of the importance of epitopes in implicating allergy, a study by Hileman et al. [138] found that high numbers of non-allergens have matching sequences of seven amino-acids with known allergens, hence such an event cannot be interpreted as an indication of an allergenic potential [138]. This notion has also been undermined as it can produce false positives in other studies [139, 140, 143–145]. Moreover, it has been reported that for any cross-reactivity to occur, in addition to the shared epitope, a high degree of similarity (between > 50–70%) is needed over a segment of 80 or greater amino acids [33, 146, 147]. As no such significant sequence identity match was found between hCas9 and Hev b 9, the epitope match identified between hCas9 and Hev b 9 can be considered as unlikely to pose an allergenic risk.

#### Literature assessment of hCas9 allergenicity

Cas9 has commonly been used in studies to evaluate allergens and immune-related diseases such as egg allergies [148], or to remove allergens from products such as soybean through site-directed mutagenesis [149] or target genes involved in allergic rhinitis [150]; or remove and functionally evaluate other allergens [151–156], with no literature studies having implicated Cas9 proteins themselves in allergenicity. Importantly, Nakajima et al. [54] reported that Cas9 was subject to rapid digestion in vitro and unlikely to cause food allergy in the case of accidental ingestion.

Human exposure to Cas9 is widespread, with acquired immune responses to Cas9 having been well documented [157–161]. Charlesworth et al. [157] detected antibodies and T cells against SpCas9 in 58% and 67% of 125 antibody donors, respectively. Wagner et al. [160] detected antibody titers against *S*.* pyogenes* antigens and SpCas9-reactive regulatory T cells in 85% and 96% of 48 donor individuals, respectively. Ferdosi et al. [158] detected anti-SpCas9 antibodies in more than 5% of 143 healthy individuals, while Simhadri et al. [159] found anti-SpCas9 antibodies in 2.5% of 248 donors. Charlesworth et al. [157] showed that donors positive for cellular activity against Cas9 were also positive for antibody activity, demonstrating a high concordance between adaptive and humoral immunity.

Moreover, a study by El-Mounadi et al. [162] outlines Cas9 sequence similarity of 23–58% to Cas9 protein present in *Streptococcus thermophilus* and *Lactobacillus plantarum,* two species of bacteria widely used as a probiotic in the production of cheese and yoghurt. Indeed, *L*.* plantarum* is frequently encountered as a natural inhabitant of the human gastrointestinal tract, as a result of it being a transient guest, introduced through diet [163]; and has been shown to contribute to inhibition of pathogenic bacteria [164]. El-Mounadi et al. [162] further demonstrated more than 80% amino acid sequence similarity detected between Cas9 from *S*.* pyogenes* and that from human commensal and pathogenic bacteria such as *Streptococcus dysgalactiae *subsp.* equisimilis, Staphylococcus aureus, Klebsiella pneumonia* and *Streptococcus canis*.

### DsRed toxicity

#### Bioinformatic assessment of DsRed toxicity

NCBI TRS and EST database searches yielded no entries with an E-value below 10. Searches of BLAST UniProt-Toxin protein databases with DsRed identified 21 entries with E-values below 10 (see Table 3). BLAST UniProt-Toxin database searches yielded three entries with significant sequence similarity of greater than 35% over more 80 or more amino acids, but all were fluorescent proteins encoded by cloning vectors, and none were toxins. The remaining identified entries showed no significant sequence alignments to DsRed.

**Table 3:** Bioinformatic search results for sequence identity with DsRed

Database	Program	Search restriction	Accession #	Organism	Description	Length (aa)	*E* value	% identity	Alignment length (aa)	Comments from manual inspection of alignment
BLAST UniProt-Toxin	BLASTP	Toxin	A0A8A1QQ63	Synthetic DNA	DsRed OS = Cloning vector tetO-AaHIT	238	1e−163	98	223	Homologous to fluorescent marker in construct
BLAST UniProt-Toxin	BLASTP	Toxin	A0A8A1QPY2	Synthetic DNA	ZsGreen OS = Cloning vector Op/Ie2-tTAV(OX4585)	247	2e−57	46	205	Homologous to fluorescent marker in construct
BLAST UniProt-Toxin	BLASTP	Toxin	A0A8A1QPH2	Synthetic DNA	ZsGreen OS = Cloning vector Hr5/Ie1-tTAV(OX5378)	247	2e−57	46	205	Homologous to fluorescent marker in construct
BLAST UniProt-Toxin	BLASTP	Toxin	A0A059PIQ0	*Aequorea victoria*	Green fluorescent protein (Fragment)	251	1e−17	25	218	Homologous to green fluorescent protein
BLAST UniProt-Toxin	BLASTP	Toxin	A0A1I9LJZ6	*Stx converting phage vB_EcoS_ST2-8624*	Uncharacterized protein	238	1e−17	25	218	Alignment identity n.s
BLAST UniProt-Toxin	BLASTP	Toxin	A0A1I9LJ85	*Stx converting phage vB_EcoS_P27*	Uncharacterized protein	262	2e−17	25	218	Alignment identity n.s
BLAST UniProt-Toxin	BLASTP	Toxin	A0A1I9LJQ6	*Stx converting phage vB_EcoS_P27*	Uncharacterized protein	262	3e−17	25	218	Alignment identity n.s
BLAST UniProt-Toxin	BLASTP	Toxin	A0A1I9LJG8	*Stx converting phage vB_EcoS_P32*	Uncharacterized protein	262	3e−17	25	218	Alignment identity n.s
BLAST UniProt-Toxin	BLASTP	Toxin	A0A1I9LJ85	*Stx converting phage vB_EcoS_P27*	Uncharacterized protein	262	3e−17	25	218	Alignment identity n.s
BLAST UniProt-Toxin	BLASTP	Toxin	A0A0P0UVZ8	*Staphylococcus aureus*	Toxic shock syndrome toxin	103	1e−04	54	41	Alignment length n.s
BLAST UniProt-Toxin	BLASTP	Toxin	A0A1M6ERQ6	*Flavobacterium terrae*	Uncharacterized protein	128	0.80	28	115	Alignment identity n.s
BLAST UniProt-Toxin	BLASTP	Toxin	F0ICN8	*Capnocytophaga *sp.* oral taxon 338 str*.* F0234*	Uncharacterized protein	128	1.9	28	115	Alignment identity n.s
BLAST UniProt-Toxin	BLASTP	Toxin	A0A2S0L9N8	*Capnocytophaga *sp.* oral taxon 878*	Phage tail protein	128	1.9	28	115	Alignment identity n.s
BLAST UniProt-Toxin	BLASTP	Toxin	L1P5W9	*Capnocytophaga *sp.* oral taxon 326 str*.* F0382*	Uncharacterized protein	128	2.0	28	115	Alignment identity n.s
BLAST UniProt-Toxin	BLASTP	Toxin	A0A172XSB0	*Chryseobacterium glaciei*	Phage tail protein	128	2.2	27	115	Alignment identity n.s
BLAST UniProt-Toxin	BLASTP	Toxin	A0A502H3U0	*Brevibacillus laterosporus*	Uncharacterized protein	287	2.4	29	82	Alignment identity n.s
BLAST UniProt-Toxin	BLASTP	Toxin	A0A2S5I5K9	*Brevibacillus laterosporus*	Toxin-like protein	356	2.4	29	82	Alignment identity n.s
BLAST UniProt-Toxin	BLASTP	Toxin	U2C560	*Capnocytophaga *sp.* oral taxon 863 str*.* F0517*	Uncharacterized protein	128	2.7	28	115	Alignment identity n.s
BLAST UniProt-Toxin	BLASTP	Toxin	A0A3G6MH72	*Chryseobacterium *sp.* G0201*	Phage tail protein	128	2.7	26	115	Alignment identity n.s
BLAST UniProt-Toxin	BLASTP	Toxin	A0A0E9WUE2	*Anguilla anguilla*	Uncharacterized protein	135	7.2	26	57	Alignment identity n.s
BLAST UniProt-Toxin	BLASTP	Toxin	A0A345CDE5	*Leclercia* sp.* W17*	Type II toxin-antitoxin system RelE/ParE family toxin	98	8.9	35	49	Alignment length n.s
BLAST UniProt-Toxin	BLASTP	Toxin	A0A5Q6S3U2	*Mumia* sp.* Z350*	Type II toxin-antitoxin system VapB family antitoxin	98	9.6	28	81	Alignment identity n.s
BLAST UniProt-Allergen	BLASTP	Allergen	A0A1Y2VHK8	*Hypoxylon *sp.* CO27-5*	Glycoside hydrolase family 128 protein	461	0.051	25	59	Below 35%/80aa cutoff
BLAST UniProt-Allergen	BLASTP	Allergen	A0A1Y2TLJ6	*Hypoxylon *sp.* EC38*	Glycoside hydrolase family 128 protein	461	0.52	28	43	Below 35%/80aa cutoff
COMPARE/AllergenOnline	COMPASS/Fasta36	Allergen	ADL32664.1	*Daucus carota*	Allergen PRP-like protein	154	0.065/1.0	23.2	125	Below 35%/80aa cutoff
COMPARE/AllergenOnline	COMPASS Fasta36	Allergen	ADL32662.1	*Daucus carota*	Allergen PRP-like protein	154	0.15/2.0	23.2	125	Below 35%/80aa cutoff
COMPARE/AllergenOnline	COMPASS/Fasta36	Allergen	ADL32665.1	*Daucus carota*	Allergen PRP-like protein	154	0.18/2.3	23.2	125	Below 35%/80aa cutoff
COMPARE/AllergenOnline	COMPASS/Fasta36	Allergen	ADL32666.1	*Daucus carota*	Allergen PRP-like protein	154	0.18/2.3	23.2	125	Below 35%/80aa cutoff
COMPARE/AllergenOnline	COMPASS/Fasta36	Allergen	ADL32663.1	*Daucus carota*	Allergen PRP-like protein	154	0.22/2.7	23.2	125	Below 35%/80aa cutoff
COMPARE/AllergenOnline	COMPASS/Fasta36	Allergen	BAB88129.1	*Daucus carota*	Allergen pathogenesis-related protein-like	154	0.22/2.7	23.2	125	Below 35%/80aa cutoff
COMPARE/AllergenOnline	COMPASS/Fasta36	Allergen	AAS75831.1	*Suidasia medanensis*	Putative group 2 allergen Sui m 2	141	0.82/7.1	25.9	58	Below 35%/80aa cutoff
COMPARE/AllergenOnline	COMPASS/Fasta36	Allergen	BAB21491.1	*Betula platyphylla*	Allergen Bet vI jap3	160	1.0/8.7	57.9	19	Below 35%/80aa cutoff
COMPARE	COMPASS	Allergen	AAS47037.1	*Prunus avium*	Allergen Pru av 1	160	1.2	24.4	131	Below 35%/80aa cutoff
COMPARE	COMPASS	Allergen	CAB06416.1	*Daucus carota*	Allergen major allergen Dau c 1/1	154	1.4	21.6	125	Below 35%/80aa cutoff
COMPARE	COMPASS	Allergen	BAA13604.1	*Daucus carota*	Allergen Dau c 1	154	1.4	21.6	125	Below 35%/80aa cutoff
COMPARE	COMPASS	Allergen	AAB01092.1	*Daucus carota*	Allergen Dau c 1	168	1.7	21.6	125	Below 35%/80aa cutoff
COMPARE	COMPASS	Allergen	CAA07320.1	*Betula pendula*	Allergen pollen allergen Betv1, isoform at1	120	1.7	52.6	19	Below 35%/80aa cutoff
COMPARE	COMPASS	Allergen	CAA07328.1	*Betula pendula*	Allergen pollen allergen Betv1, isoform at8	120	1.7	52.6	19	Below 35%/80aa cutoff
COMPARE	COMPASS	Allergen	CAB03715.1	*Daucus carota*	Allergen Dau c 1	154	1.8	20.8	125	Below 35%/80aa cutoff
COMPARE	COMPASS	Allergen	AAQ24541.1	*Blomia tropicalis*	Putative Blo t 1 allergen	333	2.4	25.4	67	Below 35%/80aa cutoff
COMPARE	COMPASS	Allergen	MANUAL2	*Blomia tropicalis*	Putative Blo t 1.02 Manual Entry	333	2.4	25.4	67	Below 35%/80aa cutoff
COMPARE	COMPASS	Allergen	CAB03716.1	*Daucus carota*	Allergen Dau c 1	154	2.7	23.8	126	Below 35%/80aa cutoff
COMPARE	COMPASS	Allergen	1B6F_A	*Betula pendula*	Allergen Chain A, Birch Pollen Allergen Bet V 1	159	2.8	52.6	19	Below 35%/80aa cutoff
COMPARE	COMPASS	Allergen	CAA04829.1	*Betula pendula*	Allergen pollen allergen, Betv1	159	2.8	52.6	19	Below 35%/80aa cutoff
COMPARE	COMPASS	Allergen	4BK6_B	*Betula pendula*	Allergen Chain B, Crystal Structure Of A Dimeri	159	2.8	52.6	19	Below 35%/80aa cutoff
COMPARE	COMPASS	Allergen	4BK7_A	*Betula pendula*	Allergen Chain A, Crystal Structure Of A Varian	159	2.8	52.6	19	Below 35%/80aa cutoff
COMPARE	COMPASS	Allergen	CAA07324.1	*Betula pendula*	Allergen pollen allergen Betv1, isoform at4	160	2.9	52.6	19	Below 35%/80aa cutoff
COMPARE	COMPASS	Allergen	CAA07323.1	*Betula pendula*	Allergen pollen allergen Betv1, isoform at3	160	2.9	52.6	19	Below 35%/80aa cutoff
COMPARE	COMPASS	Allergen	AAD26561.1	*Betula pendula*	Allergen isoallergen Bet v 1 b2	160	2.9	52.6	19	Below 35%/80aa cutoff
COMPARE	COMPASS	Allergen	CAB02160.1	*Betula pendula*	Allergen pollen allergen Bet v 1	160	2.9	52.6	19	Below 35%/80aa cutoff
COMPARE	COMPASS	Allergen	CAA07329.1	*Betula pendula*	Allergen pollen allergen Betv1, isoform at5	160	2.9	52.6	19	Below 35%/80aa cutoff
COMPARE	COMPASS	Allergen	CAB02155.1	*Betula pendula*	Allergen pollen allergen Bet v 1	160	2.9	52.6	19	Below 35%/80aa cutoff
COMPARE	COMPASS	Allergen	CAA54487.1	*Betula pendula*	Allergen Bet v 1 j	160	2.9	52.6	19	Below 35%/80aa cutoff
COMPARE	COMPASS	Allergen	CAB02156.1	*Betula pendula*	Allergen pollen allergen Bet v 1	160	2.9	52.6	19	Below 35%/80aa cutoff
COMPARE	COMPASS	Allergen	CAB02157.1	*Betula pendula*	Allergen pollen allergen Bet v 1 [Betula pe	160	2.9	52.6	19	Below 35%/80aa cutoff
COMPARE	COMPASS	Allergen	CAA33887.1	*Betula pendula*	Allergen Bet v 1—like	160	2.9	52.6	19	Below 35%/80aa cutoff
COMPARE	COMPASS	Allergen	AAD26560.1	*Betula pendula*	Allergen isoallergen bet v 1 b1	160	2.9	52.6	19	Below 35%/80aa cutoff
COMPARE	COMPASS	Allergen	AAS47035.1	*Prunus avium*	Allergen Pru av 1	160	2.9	25.2	135	Below 35%/80aa cutoff
COMPARE	COMPASS	Allergen	CAA96547.1	*Betula pendula*	Allergen major allergen Bet v 1	160	2.9	52.6	19	Below 35%/80aa cutoff
COMPARE	COMPASS	Allergen	CAA05187.1	*Betula pendula*	Allergen pollen allergen Betv1	160	2.9	52.6	19	Below 35%/80aa cutoff
COMPARE	COMPASS	Allergen	CAA07318.1	*Betula pendula*	Allergen pollen allergen Betv1, isoform at8	160	2.9	52.6	19	Below 35%/80aa cutoff
COMPARE	COMPASS	Allergen	CAA96541.1	*Betula pendula*	Allergen major allergen Bet v 1	160	2.9	52.6	19	Below 35%/80aa cutoff
COMPARE	COMPASS	Allergen	CAA54484.1	*Betula pendula*	Allergen Bet v 1 f	160	2.9	52.6	19	Below 35%/80aa cutoff
COMPARE	COMPASS	Allergen	CAA05188.1	*Betula pendula*	Allergen pollen allergen Betv1	160	2.9	52.6	19	Below 35%/80aa cutoff
COMPARE	COMPASS	Allergen	CAB02161.1	*Betula pendula*	Allergen pollen allergen Bet v 1	160	2.9	52.6	19	Below 35%/80aa cutoff
COMPARE	COMPASS	Allergen	CAA07326.1	*Betula pendula*	Allergen pollen allergen Betv1, isoform at5	160	2.9	52.6	19	Below 35%/80aa cutoff
COMPARE	COMPASS	Allergen	BAB21490.1	*Betula platyphylla*	Allergen Bet vI jap2	160	3.5	55.0	20	Below 35%/80aa cutoff
COMPARE	COMPASS	Allergen	CAA96545.1	*Betula pendula*	Allergen major allergen Bet v 1	160	3.5	52.6	19	Below 35%/80aa cutoff
COMPARE	COMPASS	Allergen	ACD65080.1	*Forcipomyia taiwana*	Allergen For t 1	118	3.9	30.2	43	Below 35%/80aa cutoff
COMPARE	COMPASS	Allergen	CBG76811.1	*Secale cereale*	Putative pollen allergen Sec c 5	202	4.3	24.4	172	Below 35%/80aa cutoff
COMPARE/AllergenOnline	COMPASS/Fasta36	Allergen	P83834.1	*Cucumis melo*	Allergen Pathogenesis-related protein (PR-1)	41	6.1/2.3	25.6	39	Below 35%/80aa cutoff
COMPARE	COMPASS	Allergen	CAA04823.1	*Betula pendula*	Allergen pollen allergen, Betv1	159	6.5	47.4	19	Below 35%/80aa cutoff
COMPARE	COMPASS	Allergen	BAB21489.1	*Betula platyphylla*	Allergen Bet vI jap1	160	6.5	47.4	19	Below 35%/80aa cutoff
COMPARE	COMPASS	Allergen	AAS47036.1	*Prunus avium*	Allergen Pru av 1	160	6.5	23.7	131	Below 35%/80aa cutoff
COMPARE	COMPASS	Allergen	CAA54482.1	*Betula pendula*	Allergen Bet v 1 d	160	6.5	47.4	19	Below 35%/80aa cutoff
COMPARE	COMPASS	Allergen	CAA07325.1	*Betula pendula*	Allergen pollen allergen Betv1, isoform at4	160	6.5	47.4	19	Below 35%/80aa cutoff
COMPARE	COMPASS	Allergen	P43180.2	*Betula pendula*	Allergen Major pollen allergen Bet v 1-G	160	6.5	47.4	19	Below 35%/80aa cutoff
COMPARE	COMPASS	Allergen	AJE61290.1	*Prunus persica*	Allergen Pru p 1	160	6.5	24.4	131	Below 35%/80aa cutoff
COMPARE	COMPASS	Allergen	CAA54489.1	*Betula pendula*	Allergen Bet v 1 l	160	6.5	47.4	19	Below 35%/80aa cutoff
COMPARE	COMPASS	Allergen	CAA05190.1	*Betula pendula*	Allergen pollen allergen Betv1	160	6.5	47.4	19	Below 35%/80aa cutoff
COMPARE	COMPASS	Allergen	CAA07319.1	*Betula pendula*	Allergen pollen allergen Betv1, isoform at1	160	6.5	47.4	19	Below 35%/80aa cutoff
COMPARE	COMPASS	Allergen	CAA05186.1	*Betula pendula*	Allergen pollen allergen Betv1	160	6.5	47.4	19	Below 35%/80aa cutoff
COMPARE	COMPASS	Allergen	COMPARE00288	*Gallus gallus*	Allergen Gal d 9	45	8.3	70.0	10	Below 35%/80aa cutoff
COMPARE	COMPASS	Allergen	COMPARE200	*Phoenix sylvestris*	alpha 1, 4 glucan synthase, partial sequence	12	8.9	71.4	7	Below 35%/80aa cutoff
COMPARE	COMPASS	Allergen	COMPARE00276	*Delonix regia*	unknown function, partial	18	9.6	100.0	6	Below 35%/80aa cutoff
COMPARE	COMPASS	Allergen	1QMR_A	*Betula pendula*	Allergen Chain A, Birch Pollen Allergen Bet V 1	159	9.8	37.0	46	Below 35%/80aa cutoff
COMPARE	COMPASS	Allergen	CAA96542.1	*Betula pendula*	Allergen major allergen Bet v 1	160	9.9	27.6	116	Below 35%/80aa cutoff
COMPARE	COMPASS	Allergen	CAD10374.1	*Castanea sativa*	Putative ypr10	160	9.9	21.9	105	Below 35%/80aa cutoff

n.s.: not significant; 35%/80aa cutoff: alignments to allergens are considered significant when at least 35% identical over a segment of 80 or more amino acids

#### Literature assessment of DsRed toxicity

The fluorescent protein DsRed was originally isolated from *Dicosoma* spp. and is part of a broader family of intrinsically fluorescent proteins represented by Green Fluorescent Protein (GFP) [165]. GFP is a stable and soluble monomer, which is brightly fluorescent green [166]. DsRed is a tetramer and vibrantly fluorescent red with an emission wavelength of 583 nm [167]. Its synthesis occurs via an extended form of the GFP synthesis pathway, and a single amino acid replacement can convert GFP chromoproteins into a DsRed‐like fluorescent proteins [168]. Based on the homology between DsRed and GFP proteins, GFP has been used to assess the toxicity and allergenicity of the wider GFP family of proteins, including DsRed. In that regard, Richards et al. [169] found that ingestion of GFP in rodents did not affect growth, food intake, or relative weight of organs. Moreover, GFP was rapidly digested in simulated gastric fluid and found to possess insignificant levels of homology with known allergens.

In whole cell labelling experiments, moderate to high levels of intracellular expression of some variants of DsRed can be cytotoxic under in vitro conditions. For example, Strack et al. [170] found that retroviral expression of some variants of DsRed can compromise the viability of transfected HeLa cells in vitro. Retroviral-mediated expression of DsRed-Express fluorescent protein can also interfere with the viability or self-renewal of in vitro with cultures of Hematopoietic Stem Cells [170, 171]. The DsRed-Express variant has more rapidly maturing fluorescence, enhanced solubility, reduced green emission, and faster maturation rate than the original DsRed. By contrast, Strack et al. [170] also found that another variant, DsRed-Express2, had no such effect. Matsushima et al. [172] developed an in vivo imaging system using DsRed in mice to observe interleukin production in the skin, with no toxicity reported.

Nordin et al. [173] found that oral ingestion of transgenic *Aedes aegypti* expressing DsRed had no effects on life table parameters of all life stages of *Toxorhynchites splendens* and *Tx*.* amboinensis*. Following ERAs, including assessments of toxicity and allergenicity, environmental releases of these transgenic mosquitoes were approved by regulatory authorities in Brazil and Malaysia and the USA [174–178]. Likewise, the Dutch GMO Office considered there were negligible risks to human health and the environment from releases of these transgenic mosquitoes expressing DsRed [179]. In addition, DsRed is genetic element reference record in the Biosafety Clearing House of the Cartagena Protocol on Biosafety of the Convention on Biological Diversity (https://bch.cbd.int/en/database/BCH-GENE-SCBD-101476-6), supporting a history of its safe use in GMO applications.

### DsRed allergenicity

#### Bioinformatic assessment of DsRed allergenicity

NCBI TRS and EST database searches yielded no entries with an E-value below 10. Searches of BLAST Uniprot-Allergen protein databases yielded two entries with E-values below 10 but neither had significant sequence alignments (see Table 3). Using the COMPASS to search the COMPARE database, 64 entries were identified with E values below 10, but none had 35% or more sequence identity with DsRed over 80 or more amino acids. Using FASTA36 to search the AllegenOnline database, nine entries were identified with E values below 10, but none had 35% or more sequence identity with DsRed over 80 or more amino acids. For 80 sliding and 8-mer searches in COMPARE and AllergenOnline, no significant sequence alignments were identified for DsRed.

#### Literature assessment of DsRed allergenicity

No studies were found in which DsRed was implicated in allergenicity. In a single study by Matsuoka et al. 2012, where transgenic *Anopheles stephensi* mosquitoes were created to express DsRed in saliva, authors demonstrated repeated feedings inducing an anti-DsRed antibodies in mice [180]. When having “immunized” mice with injected DsRed, upon exposure to bites from the transgenic DsRed positive mosquito, the mice produced a higher titre of antibody to DsRed, suggesting that the transgenic mosquito bites acted as a “booster” effect.

### *Discosoma* spp. toxicity

#### Literature assessment of *Discosoma* spp. toxicity

Literature searches of *Discosoma* found no evidence from previous studies of potential toxicity. Long et al. [181] reported that high levels of expression of DsRed from *Discosoma* did not affect cell morphology, developmental potential or viability and fertility of mice. Barbier and Damron [182] reported no changes in viability or growth fitness in bacteria expressing DsRed.

### *Discosoma* spp. allergenicity

#### Literature assessment of *Discosoma* spp. allergenicity

For the *Discosoma* species, no evidence from previous studies was found indicating a potential for allergenicity.

### *Streptococcus pyogenes* toxicity

#### Literature assessment of *S*.* pyogenes* toxicity

There are numerous toxic effects associated with various components of *S*.* pyogenes* such as excretory toxins, cell wall components or superantigens, many of which work in concordance with one another and none of which have been directly attributed to Cas9 expression or activity. These components are outlined in more detail in Additional file 2: Tables S1, S2 and S3.

### *Streptococcus pyogenes* allergenicity

#### *Literature assessment of S*.* pyogenes allergenicity*

*Streptococcus pyogenes* components can elicit immune responses including acute reactivation or induction of arthritis [183–185]; hyperinflammatory responses [186, 187] as well as one study showing evidence of anaphylactoid response limited to rabbits only, although this effect was not observed in mice [188]. However, none of these have been directly attributed to Cas9.

### T1 gRNA allergenicity and toxicity

#### Bioinformatic assessment of T1 gRNA

No significant similarities with an E value below 0.01 were found between the T1 gRNA and miRNAs from human, *Anopheles,* or any other species in the miRbase database.

#### Literature assessment of RNA toxicity and allergenicity

Kim et al. [189] showed that gRNAs can trigger RNA-sensing, type 1 interferon immune responses in cell cultures leading to cytotoxicity. Moreover, it is known that there are numerous short RNAs present in wild type mosquito saliva, which likely get transferred into human tissues during biting [190], which could, therefore, theoretically lead to immune responses. However, human intradermal and intramuscular exposure to RNA from therapeutic interventions such as mRNA-based vaccinations against COVID-19 have a proven safety profile for use in humans [191–193]. Moreover, RNA is subject to fast breakdown in vivo and does not integrate into the genome [194] and is only transiently expressed if at all [195]. Indeed, several therapies using in vitro transcribed mRNA are currently being actively investigated in clinical trials and have demonstrated excellent tolerability and safety indicating that mRNA therapies as a class of intervention have no inherent risks [196, 197].

## Discussion

### ***Hazard characterization of toxicity and allergenicity from dsxF***^***CRISPRh***^

In bioinformatic assessments of transgenic products expressed from the *dsxF*^*CRISPRh*^ transgene, there was no significant amino acid similarity between DsRed and known allergens or toxins. hCas9 showed no evidence of identity with proteins known to be toxic to eukaryotes. While bioinformatic searches did identify homology between hCas9 and the VapC protein, a weight of evidence approach was applied in a tiered evaluation of hCas9 toxicity in humans and livestock [12–14]. Firstly, in terms of mode of action, VapC is restricted to prokaryotes via its inhibitory effects on mRNA translation, and thus cell growth, specifically in bacteria. Secondly, in terms of amino acid sequence similarity, of the 117 amino acids of VapC [42], 33 and 73 align within the HNH and RuvC-III domains found between amino acids 805-964 and 965-1141 of hCas9, respectively [43] (see Fig. 1). However, VapC does not show any sequence similarity to RuvC-I, alpha-helical lobe, RuvC-II, topo-homology and C-terminal domains found in Cas9 [43]. The two proteins therefore only share homology to a minority of the total Cas9 protein. Thirdly, in terms of history of safe use of the protein or source organism, VapC shows sequence identity to a wide range of Cas9 homologues in heterogenous bacterial species that are widely distributed throughout nature [44]. Fourthly, in terms of probability and level of exposure to any potential toxicity, the expression of Cas9 in *dsxF*^*CRISPRh*^ mosquitoes is controlled by the germline promoter *zpg* and is therefore not expected to be expressed in the saliva of *dsxF*^*CRISPRh*^ transgenics [7] so that any exposure via this route would be negligible. Moreover, the levels of Cas9 transgenic protein in any given individual *dsxF*^*CRISPRh*^ mosquito would be insignificant in terms of biomass within the environment [55]. Hence, applying a weight of evidence approach, it was concluded that the sequence similarity identified between VapC and Cas9 required no further investigation to support the conclusion that the risk of toxicity from Cas9 expression in *dsxF*^*CRISPRh*^ transgenics is negligible.

Bioinformatic analysis of the T1 gRNA expressed in *dsxF*^*CRISPRh*^ GMMs indicated no sequence similarity to known microRNAs from other species, including humans and *Anopheles*. Coupled with literature assessment demonstrating low risk from intradermal exposure to RNA, there was no evidence to suggest that the presence of the gRNA could be toxic or allergenic, for example, by interfering host gene expression.

Most of the literature identified from searches of Cas9 and DsRed described in vitro investigations of gain or loss of function of heterologous proteins, development of fluorescent reporter systems, assay development or method validation, typically providing no direct evidence indicating toxicity or allergenicity of the transgenic proteins themselves. In assessments of toxicity and allergenicity of source organisms, while there was no evidence from previous studies of allergenicity or toxicity associated with *Discosoma* spp., *S*.* pyogenes* showed some evidence of toxicity, as well as allergenicity, but this was not associated directly with Cas9 activity.

The strength of evidence supporting conclusions on allergenicity or toxicity of transgenic products in humans from in vitro*, *in vivo and clinical studies was an important consideration in literature evaluations. Results from in vitro studies may not necessarily reliably translate into the in vivo biological responses of organisms. For example, for DsRed, expression of some of its variants can result in cytotoxicity in some cell types in vitro. However, in several in vivo transgenic animal models, expression of the DsRed variant does not result in toxicity [172, 198, 199]. For Cas9, intracellular, cell-specific in vitro toxicity has been observed, as well as toxicity when Cas9 is expressed at high levels in vivo [81, 82, 105]. Importantly, Garrood et al. [106] reported that Cas9 off-target mutagenic effects could not be detected in the genome of *dsxF*^*CRISPRh*^ GMM. This is consistent with finding that Cas9 constitutive intracellular expression can be toxic to algae, fungi and parasitic organisms [98–100], but its transient expression in these organisms does not produce toxicity. In one in vivo case, zebrafish muscle fibre toxicity concomitant with expression of Cas9 was not directly attributed to Cas9 [101]. In another study looking at nucleotide insertions and indels in vivo, Cas9 knock-in mice exhibited only low levels of indels [200]. However, there were also numerous in vitro as well as in vivo studies of Cas9 providing no evidence of toxicity. Moreover, recent clinical trials involving Cas9 that target disparate organs of the body indicate a lack of any significant associated toxicity [122, 125, 126, 128, 132].

Moreover, toxicity was commonly absent from Cas9 in vivo studies and, where it was detected, this was typically associated with high levels of Cas9 expression or concentrations in vivo and absent at lower concentrations [68, 105, 108–110, 117, 200]. Furthermore, Cas9 has been administered in vivo to patients in a number of clinical trials without significant safety concerns [97, 128, 132] This evidence of safe human therapeutic exposure to Cas9, coupled with bioinformatic analyses finding no significant sequence identity matches between hCas9 and any toxic proteins, indicates that exposure to hCas9 from *dsxF*^*CRISPRh*^ GMMs is unlikely to cause toxicity in vivo in humans or other mammals.

In bioinformatic searches, hCas9 was also found to share a single eight amino acid epitope within the latex allergen Hev b 9, from *Hevea brasiliensis* [201, 202]. This contrasts with the results of Nakajima et al. [54]. Although they found one epitope to the food allergen serine carboxypeptidase 2 in *Triticum aestivum* in a soybean codon-optimized Cas9, they did not identify the Hev b 9 epitope in their searches with human codon-optimized Cas9. They used the AllergenOnline database, as was the case in this study, and the amino acid sequence of hCas9 that was used in this study was also used by Nakajima et al. [54]. It may be that this epitope has only been identified or deposited in databases since the searches of Nakajima et al. [54], which would have occurred at least five years before our searches. In any event, the present study identified the single eight amino acid epitope within the latex allergen Hev b 9 from searches of both COMPARE and AllergenOnline databases with hCas9 amino acid sequence. However, it has been reported that for any cross-reactivity to occur, in addition to the shared epitope, a high degree of similarity (between > 50 and 70%) is needed over a segment of 80 or greater amino acids [33, 146, 147], and no such significant sequence identity match was found between hCas9 and Hev b 9. The single epitope match identified between hCas9 and Hev b 9 can therefore be considered as unlikely to pose an allergenic risk. This conclusion is reinforced by the evidence of widespread exposure of humans and other animals to bacteria expressing Cas9 variants. Acquired immune responses to Cas9 are widespread in humans and have been well documented, with antibodies and T cells against SpCas9 detected in 58% and 67% of donors, respectively [157–160].

### ***Exposure characterization of toxicity and allergenicity from dsxF***^***CRISPRh***^

Key considerations in the ERA for toxicity and allergenicity of transgenic proteins involve routes and levels of exposure. Although GMMs would not be actively consumed by humans, accidental incidental ingestion or inhalation of mosquitoes, or their body parts, may occur but is still likely to be a negligible source of human exposure to transgenic proteins. Because of the restricted temporal and spatial expression of the promoter driving expression of Cas9, its expression should be restricted to the germline in males and females from late pupal stages; and the levels of Cas9 transgenic protein in any given individual mosquito would be negligible in terms of biomass [55]. Even if accidental ingestion of minimal amounts of material were to occur, Cas9 is subject to pepsin digestion [54] and, therefore, exposure in the body to Cas9 through accidental ingestion of a GMM, would be negligible.

However, female mosquitoes require mammalian, in some cases preferentially human, blood to produce viable eggs and offspring. Therefore, the substantive route of human exposure to mosquito proteins is intradermal via the blood-feeding of female mosquitoes and local allergic reactions to saliva from mosquito bites, which have been observed in humans [203–205]. In *dsxF*^*CRISPRh*^ GMMs, homozygous females are unable to bite, limiting the risk of exposure to transgenic products via saliva from the bites from heterozygous females. In addition, Cas9 is expressed under the control of the germline promoter *zpg* and DsRed under the control of an ocular and nervous system promoter *3X3P* [7]. Therefore, neither protein should be expressed in the salivary glands, nor be present in the saliva, of *dsxF*^*CRISPRh*^ GMMs. By contrast, in the *dsxF*^*CRISPRh*^ transgene, the T1 gRNA is under the control of the ubiquitous and constitutive *U6* promoter. It is, therefore, expected that the T1 gRNA transgenic product would be present in the saliva of these transgenic mosquitoes.

The nuclease expressed from the *dsxF*^*CRISPRh*^ transgene is a human codon-optimized version of Cas9 that originates from *S*.* pyogenes*¸ a dominant member of Group A *Streptococci* (GAS) [7, 206]. *Streptococcus pyogenes* is commonly found throughout the world in the throat, skin and anogenital tract of humans, including infants and healthy individuals. Approximately 20% of children are chronic carriers of GAS [207]. Shaikh et al. [208] reported that prevalence of Group A *Streptococci* in children presenting with pharyngitis was 37%, compared with 12% in those with no symptoms. Another study reported oropharyngeal colonization by *S*.* pyogenes* in 9.6% of young adults [209]. Carapetis et al. [210] have reported a global disease burden of over 111 million cases of GAS pyoderma, and over 616 million incident cases per year of GAS pharyngitis. Human exposure to *S*.* pyogenes* Cas9 (SpCas9) is thus common and frequent, with development of acquired immune responses [157–160]. These studies provide evidence of widespread pre-existing human exposure to the Cas9 protein. Moreover, in food toxicity evaluations, if a modified protein retains biological function, and this function is found in related proteins with a history of safe use in food, and the exposure level is similar to functionally related proteins, then the modified protein could also be considered to be ‘as-safe-as’ those that have the history of safe use [211]. It has been proposed that preexisting immunity to Cas9 may decrease its efficacy in vivo in a therapeutic context [212, 213] however a population’s prior exposure to Cas9 orthologues from pathogenic bacteria such as *S*.* thermophilus* may prompt effective recognition, targeting by cytotoxic T cells and ultimately clearance of any cells expressing Cas9 [213].

These results indicate generation of an immune response to SpCas9, wherein subsequent exposure to the protein would result in antibodies binding to and rendering the protein inactive, or formation of immune complexes that are actively cleared by the circulation [213], which would be favourable in case of GMMs expressing Cas9 protein, or should the protein itself be exposed to the immune system. The conclusion that a single epitope match between hCas9 and Hev b 9 is unlikely to pose an allergenic risk is therefore further reinforced by the evidence of widespread exposure of humans and other animals to bacteria expressing Cas9 variants.

### ***Evaluation of potential toxicity or allergenicity from dsxF***^***CRISPRh***^*** mosquitoes to organisms in the environment other than humans***

Although bioinformatic and literature analyses of potential toxicity and allergenicity of *dsxF*^*CRISPRh*^ transgenic products focused primarily on human health protection goals, they are nonetheless also relevant to organisms in the environment other than humans. Five of the 46 identified plausible pathways to potential harm from field releases of mosquitoes in West Africa could result in negative impacts on livestock, animals or other non-target organisms in the environment as a result of toxicological effects [17]. As with pathways involving toxicological or allergenic effects on humans, a tiered approach based on weight-of-evidence has been applied to evaluation of potential toxicity or allergenicity from *dsxF*^*CRISPRh*^ mosquitoes to organisms in the environment other than humans [12, 13, 16, 214]. This allows the procurement of evidence only when it might directly reduce uncertainties in the ERA to prevent unwarranted, wasteful or uninformative investigations [214].

In terms of mode of action, many of the previous studies identified here draw on toxicological impacts Cas9 and DsRed transgenic proteins in animals. For example, expression of Cas9 in vivo in mice resulted in no off-target mutagenesis or evidence of toxicity [68] and oral ingestion of transgenic *Ae*.* aegypti* expressing DsRed had no effects on life table parameters of all life stages of *Tx*.* splendens* and *Tx*.* amboinensis* [173]. Hence, hazard assessments of toxicological effects and mode of action from *dsxF*^*CRISPRh*^ on humans should have relevance to impacts on other animals in the environment. Importantly, VapC toxicity is restricted to prokaryotes via specific inhibition of bacterial mRNA translation. Considering amino acid sequence similarity, although VapC aligns within the HNH and RuvC-III domains of hCas9, it does not show any sequence similarity to RuvC-I, alpha-helical lobe, RuvC-II, topo-homology and C-terminal domains [43] so that the two proteins therefore only share homology to a minority of the total Cas9 protein.

In terms of probability and level of exposure to any potential toxicity, as the *dsxF*^*CRISPRh*^ transgene is expected to introgress into sibling species of the *An*.* gambiae* complex that are zoophilic [17, 215], it is possible that some non-human animal could be exposed to transgenic products via biting as *dsxF*^*CRISPRh*^ mosquitoes attempt to obtain blood meals. However, as is the case for human exposure, neither Cas9 nor DsRed is expected to be present in the saliva of *dsxF*^*CRISPRh*^ mosquitoes; non-human animal exposure via this route would therefore be negligible. However, even though levels of Cas9 transgenic protein in any given individual *dsxF*^*CRISPRh*^ mosquito would be insignificant in terms of biomass within the environment [55], ingestion of *dsxF*^*CRISPRh*^ mosquitoes could be a more substantive route of exposure for predator or scavenger non-target organisms in the environment than for humans. Foraging theory predicts that predation of adult mosquitoes would be energetically unfavourable for large vertebrates, such as birds or bats [55, 216]. Species of the *An*.* gambiae* complex also tend to favour oviposition in aquatic habitats such as temporary and shallow puddles, irrigation channels of rice paddies or slow-moving water by riverbanks that are less likely to support colonization by many vertebrate predators [55]. Consumption of aquatic stages of these mosquitoes tends to be opportunistic and by generalist predators. Consequently, there is no evidence that any species preys exclusively on mosquitoes of the *Anopheles* genus [55]. Hence, exposure of generalist predators to *dsxF*^*CRISPRh*^ transgenic products is expected to be low, but not negligible. Nonetheless, considering the “history of safe use of the protein or source organism”, as is the case for humans, any organisms in the environment would also be exposed throughout their lifetimes, including via consumption, to a wide range of Cas9 homologues in heterogenous bacterial species that are widely distributed throughout nature [44].

Applying a weigh-of-evidence approach to the above evidence, therefore, any additional contribution from transgenic Cas9 to overall exposure to from Cas9 transgenic proteins should be considered as negligible, as would be the toxicological or allergenic hazard. Hence, overall risk of toxicity or allergenicity from releases of *dsxF*^*CRISPRh*^ mosquitoes to organisms in the environment other than humans would be negligible.

## Conclusions

Previously, the first stage of an ERA, problem formulation, was conducted to identify potential harms from simulated releases of *dsxF*^*CRISPRh*^ in West Africa as a malaria vector control intervention [17]. That analysis identified 46 plausible pathways to potential harm, nine of which could be evaluated by testing the risk hypothesis that *dsxF*^*CRISPRh*^ transgenic products would not cause increased allergenicity or toxicity. The present study formed part of the next stages of that ERA where those risk hypotheses were tested. Amino acid sequences of DsRed and hCas9 were interrogated against those of known toxins or allergens from four different bioinformatic databases and literature was examined for any evidence of toxicity or allergenicity of the transgenic products themselves, or of the donor organisms from which they were originally derived. Potential exposure to *dsxF*^*CRISPRh*^ transgenic proteins from environmental releases was assessed as negligible. Nonetheless, Cas9 nuclease activity can be toxic to some cell types in vitro and hCas9 was found to share homology with the prokaryotic toxin VapC. However, there was no evidence from previous studies indicating a risk of toxicity to humans and other animals. hCas9 was also found to contain an 8-mer epitope shared with the latex allergen Hev b 9. However, the full amino acid sequence of hCas9 was not homologous to any known allergens. Combined with a lack of evidence in the literature of Cas9 allergenicity, this indicated negligible risk of allergenicity from hCas9. No matches were found between the T1 gRNA and microRNAs from *Anopheles* or humans. Therefore, taking into account the weight of evidence on transgenic products (i) modes of actions (ii) sequence similarity to known or putative toxins or allergens, (iii) history of safe use, or the safe use of source organisms, and (iv) probability and level of exposure, there was no convincing evidence from previous studies to suggest that transgenic products expressed from *dsxF*^*CRISPRh*^ would be allergenic or toxic. These data supported acceptance of the risk hypothesis that *dsxF*^*CRISPRh*^ transgenic products would not cause increased allergenicity or toxicity, allowing the rejection of nine of a total of 46 plausible pathways to potential harm, namely pathways 1, 9, 11, 12, 16, 17, 28, 31 and 44 [17]. Hence, environmental releases in West Africa of the *dsxF*^*CRISPRh*^ population suppression gene drive for malaria vector control should not result in any increased allergenicity or toxicity in humans and animals. These results should also inform evaluations of the field use of other GMM strains for control of vectors of malaria and other infectious diseases, as well as pre-clinical risk assessments of in vivo clinical applications of CRISPR-Cas9.

### Supplementary Information

**Additional file 1.** Searches for homologues of F. necrophorum VapC in other species.

**Additional file 2.** Molecular components of S. pyogenes associated with pathogenicity.

## Data Availability

Non applicable.

## Abbreviations

aa: Amino acids
COMPARE: Comprehensive Protein Allergen Resource Database
COMPASS: COMPare Analysis of Sequences with Software Program
DSB: Double-stranded break
Ig: Immunoglobulin
EFSA: European Food Safety Authority
EMBL-EBI: European Molecular Biology Laboratory’s European Bioinformatics Institute
ERA: Environmental risk assessment
EST: Expressed sequence tags
GAS: Group A *Streptococci*
GM: Genetically modified
GMO: Genetically modified organism
GMM: Genetically modified mosquito
hCas9: Human codon-optimized version of CRISPR-Cas9 endonuclease, derived from SpCas9
HDR: Homology directed repair
MMEJ: Microhomology-mediated end joining
NCBI: National Centre for Biotechnology
NHEJ: Non-homologous end joining
NRLA: Natural rubber latex allergy
OECD: Organization of Economic Cooperation and Development
RNP: Ribonucleoprotein
SIB: Swiss Institute of Bioinformatics and Protein Information Resource
SpCas9: Cas9 of *S*.* pyogenes*
TRS: Transcript reference sequences
WHO: World Health Organization
