# Supplementary material for: Bioinformatic and literature assessment of toxicity and allergenicity of a CRISPR-Cas9 engineered gene drive to control Anopheles gambiae the mosquito vector of human malaria
Source: Malar J. 2023 Aug 14;22:234. doi: 10.1186/s12936-023-04665-5 (PMC10426224; doi:10.1186/s12936-023-04665-5)
Supplement: Supplementary file 2 — Additional file 2. Molecular components of S. pyogenes associated with pathogenicity. [file 12936_2023_4665_MOESM2_ESM.docx]

| **Table S1. Components of *Streptococcus pyogenes* found to demonstrate pathogenic impact *in vivo* in humans or animals, from literature reviews performed on or before 31^st^ March 2022** | |
| --- | --- |
| Toxic component | Studies demonstrating effects (*in vivo*) |
| Streptolysin O (SO) | Zhu *et al..* 2017 found that isogenic mutants lacking SLO were impaired in the ability to cause necrotising myositis [1]. In an *in vivo* study showing evidence of hemolytic action, Kaplan *et al.* 1976 showed lipids extracted from rabbit skin block the hemolytic activity of SO as well as the neutralising antibody response to this antigen [2]. Shanley *et al.* 1996 showed intra-tracheal administration of two types of SO, independently caused lung injury, as measured by pulmonary vascular leak, augmented by streptococcal cysteine protease [3]. Bryant *et al.* 2005 showed SO induced coaggregation of platelets and neutrophils in a rat model [4]. Brosnahan *et al.* 2009 demonstrated enhanced penetration of SPE A, through SO across porcine vaginal mucosa, causing damage to the uppermost layers of vaginal tissue [5]. In a study by Chiarot *et al.* 2013, a streptococcal strain expressing a double-mutated SO, a drastic reduction in virulence as well as a diminished capacity to kill immune cells recruited at the infection site was observed [6]. Abe *et al.* 1997 showed that repeated intracutaneous injections of SPEs A and C along the intermediate auricular artery of the rabbit ear produced subacute type arteritis [7]. Limbago *et al.* 2000 found that mice infected with an SO mutant exhibited a significant decrease in mortality rates compared to mice infected with wild-type GAS, indicating that SO plays an important role in GAS virulence [8]. |
| Streptolysin S (SS) | In a study by Fontaine *et al.* 2003, comparisons over a range of infective doses showed that both SO and SS contributed to the early stages of infection and to the induction of necrotic lesions in the murine s.c. model [9]. |
| Streptococcal pyrogenic exotoxin (SPE), Superantigen | Smoot *et al.* 2002 found that SPEs L and M were both toxic in models of animal toxic shock [10]. Murai *et al.* 1987 found induction of various pathophysiological changes after intravenous dose of SPE into rabbits [11]. Kamezawa *et al.* 1989 found evidence of mitogenic activity through blastogenic response of rabbit lymphocytes to SPE A [12]. In a study by Lee *et al.* 1989, toxins were delivered through mini-osmotic pumps implanted subcutaneously in rabbits, with SPE A found to be significantly more toxic to rabbits than C [13]. Peptide antagonists against SPE A and C protected mice against lethal challenges with components, rescuing mice undergoing toxic shock, in a study by Arad *et al.* 2001 [14]. In a study by Saouda *et al.* 2001, SPE B positive variants resulted in severe local tissue damage [15]. Hanna and Watson *et al.* 1965 and again in 1968 found that intravenous injection of 1.0 ml of SPE produced by a strain of type 18, group A streptococcus caused a prolonged (at least 24 hr) depression of reticulo-endothelial function [16, 17]. Kamezawa *et al.* 1990 showed that highly purified SPE A caused increased vascular permeability, erythema, and leukocyte emigration when injected into the skin of rabbits [18]. Akiyama *et al.* 1992 found that mice made tolerant to SPE by neonatal inoculation with SPE emulsified in incomplete Freund's adjuvant demonstrated early thrombocytopenia followed by thrombocytosis [19]. Kuo *et al.* 1998 showed that SPE B mutants caused less mortality and tissue damage than protease-positive strains when inoculated into BALB/c mice via air pouch [20]. Lukomski *et al.* 1999 demonstrate that SPE B expression contributes to soft tissue pathology, including necrosis, and is required for efficient systemic dissemination of the mouse model [21]. |
| M protein | M protein was deemed responsible by Schmidt *et al.* 1993, for high virulence and capacity to bind fibrinogen, as well as correlation with killing chicken embryos [22]. Soehnlein *et al.* 2008 showed that M1 protein injected intravenously into mice induced neutrophil accumulation in the lung, increased vascular permeability and acute lung damage [23]. Zhang et al 2012 found that an enzyme inhibitor (of an enzyme that regulates anti-inflammatory effects) markedly decreased M1 protein-induced accumulation of neutrophils, edema formation and tissue damage in the lungs of mice [24]. |
| Polysaccharide cell wall extracts or whole | Peritoneal macrophages from Fischer 344 rats were cytotoxic for mouse fibroblasts after in vitro phagocytosis of non-biodegradable group A streptococcal cell walls in a study by Smialowicz *et al.*, 1977 [25]. Schwab *et al.* 1959 found that injection of sonic-disrupted bacteria causes chronic multi-nodular lesions in the dermal connective tissue of rabbits [26], and Roberson *et al.* 1960 showed antibodies directed to the polysaccharide C were able to neutralise this toxic activity (fibrinoid necrosis) [27]. Cromartie, 1977 showed that whole killed peptidoglycan cells were able to induce arthritis in rats, measured through erythema, edema of periarticular tissues, and enlargement and distortion or ankylosis of joints [28]. Haraoui *et al.* 1985 demonstrated that retinoid was able to suppress acute and chronic responses (including arthritis) of the rat, to cell wall extract [29]. Woolverton *et al.* 1989 found induction of transient petechial haemorrhage of the rat intestine which appears within 2-3mins and resolves by 48-72hours after injection with peptidoglycan-polysaccharide polymers [30]. Muller-Alouf *et al.* 1992 showed that lipopolysaccharide (LPS) was able to induce different amounts of IL-6 and IL-3 in mice [31]. Shanley *et al.* 1996 showed intra-tracheal administration of either Streptococcal Cell Wall extract (SCW) caused lung injury, as measured by pulmonary vascular leak [3]; SCW induced neutrophil accumulation and appearance of IL-1beta and TNF-alpha. These effects were augmented by Streptococcal Cysteine Protease (SCP). Fuseler *et al.* 1997 showed that A single injection of peptidoglycan polysaccharide intraperitoneally into rats was able to induce acute joint inflammation. TNF and serum IL-1 preceded increase in IL-6 and NO production [32]. Production of these proinflammatory cytokines and NO-preceded bone erosion and osteoclast activity. Yokoi *et al.* 1997 showed endotoxemia-induced acute respiratory distress syndrome and (ARDS)-like lung injury were induced through administration of sub-lethal dose of lipopolysaccharide (LPS), intravenously [33]. Roberson *et al.* 2016 found that intravenous injection of cell wall mixture into rabbits caused them to become very weak with symptoms such as hyperemia of the eye and diarroea. One rabbit died [34]. When similar concentrations were injected into mice (5mg), many mice died. Schwab *et al.* 1962 found that microscopic features on dermal connective tissue of rabbits through after intradermal injection with a complex of group-specific C polysaccharides indicate that the tissue changes are a result of direct toxic action on connective tissue elements [35]. Schwab *et al.* 1982 were able to show that experimental arthritis developed in rats injected intraperitoneally with aqueous suspensions of peptidoglycan-polysaccharide (PG-PS) complexes isolated from group A streptococcal cell walls [36]. The quantity of cell wall per joint at day 3 correlated with the severity of joint disease. Fox *et al.* 1984 showed that PG-PS elicited chronic inflammation characterized by mononuclear and lymphocyte infiltration and severe necrosis of the retina of rabbits [37]. Hamada *et al.* 1985 showed that lipoteichoic acid (LTA) induced macrophage activation which resulted in tumor cytotoxicity in mice [38]. Wells *et al.* 1986 show that a single injection of an aqueous suspension of peptidoglycan-polysaccharide (PG-PS) when injected intraperitoneally into Lewis rats induced a self-limiting bilateral uveitis with associated perpetuating polyarthritis [39]. Leon *et al.* 1987 showed that mice injected repeatedly, intra-peritoneally or intravenously, for approximately 1 month with a total of 1.04 mg lipoteichoic acid from a nephritogenic strain of *S. pyogenes* lost weight [40]. Analysis by electron microscopy revealed that they also exhibited extensive kidney changes in basement membrane morphology which resembled, in part, those observed in human post-streptococcal glomerulonephritis [40]. When Kita *et al.* 1999 conducted a rat middle ear and nasal perfusion with LTA, several cytokines were induced *in vivo* and play an important role in the inflammatory cascade of the middle ear [41]. Middelveld *et al.* 2000 showed that PepG, a peptidoglycan, and LTA act synergistically to cause respiratory failure and septic shock in the pig [42]. |
| Erythrogenic toxins (ET) | Muller-Alouf *et al.* 1992 showed that ET type A was able to induce different amounts of IL-6 and IL-3 in mice [31]. Abe *et al.* 1997 showed Intracutaneous injections of toxins in rabbit ears produced subacute type arteritis, characteristic of lymphocytic infiltration, simulating Kawasaki disease angiitis [7]. |
| NADase | Zhu *et al.* 2017 found that isogenic mutants lacking NADase were impaired in the ability to cause necrotising myositis [1]. |
| Regulatory elements | Cho *et al.* 2013 show that GdpP influences the biogenesis of SPE B, the major secreted cysteine protease, at a post-translational level, susceptibility to the beta lactam antibiotic ampicillin, and is necessary for full virulence in a murine subcutaneous infection model [43]. |
| Other secretory proteins | Nandakumar *et al.* 2007 demonstrated EndoS (Endo-beta-N-acetylglucosaminidase) hydrolyses a glycan of human IgG [44]. Authors analysed arthritogenicity of EndoS-treated collagen type II-specific mouse mAb *in vivo.* Endoglycosidase treatment inhibited induction of arthritis in mice. Cysteine protease, in a study by Honda-Ogaw *et al.* 2013, was shown to degrade complement [45]. |
| Unverified toxin | Yoshizawa *et al.* 1997 showed that upon administration of pre-absorbing antigen to rabbits for 8 days, proliferative glomerulonephritis with exudative changes was seen, similar to those seen in acute post-streptococcal glomerulonephritis (APSGN) [46]. |

| **Table S2. Components of *Streptococcus pyogenes* found to demonstrate evidence of pathogenic impact *in vitro*, from literature reviews performed on or before 31^st^ March 2022** | |
| --- | --- |
| Toxic component | Studies demonstrating effects (*in vitro*) |
| Streptolysin O (SO) | Hurley *et al.* 2016 evaluated SO through high dose application to the myeloblastic cell line HL60 confirm toxicity, resulting in complete lysis [47]. Van Epps and Andersen, 1974 demonstrated SO-treated human peripheral leukocyte cell preparations were markedly suppressed when compared with controls [48]. Cholesterol (a known inhibitor of SO) treatment of the SO preparation blocked the hemolytic activity. Miller *et al.* 2018 found that B-cell plasma membranes damaged by SO undergo a repair process that removes the pores via a lipid raft mediated endocytosis [49]. Bhakdi *et al.* 1985 explored the method of membrane damage by SO, through the C5b-9 complement complex [50]. After human monocyte stimulation with SO, Hackett and Stevens, 1992 found it a potent inducer of TNF-alpha, and synergistically regulated Interleukin-1beta production [51]. Engel *et al.* 1995 examined effects of SO on permeability of round window membrane of the ear, resected, *in vitro* – SO evoked permeability defects dose-dependently on the round window membrane [52]. Ginsburg *et al.* 1998 showed that cell-killing was seen to be synergistic when SO combined with oxidants and proteinases when applied to monkey kidney epithelial cells and rat heart cells [53]. Sierig *et al.* 2003 showed that *in vitro* bacterial SO resulted in lysis of human keratinocytes and polymorphonuclear leukocytes [54]. Chandrasekar *et al.* 2016 found that SO, working in tandem with NAD+ glycohydrolase accelerated death response in epithelial cells [55]. Nanosponge administration protected human neutrophils, macrophages and keratinocytes against SO-mediated cytotoxicity in a study by Escajadillo *et al.* 2017 [56]. Sogawa *et al.* 2018 found that water soluble polyphenols contained in olives have inhibitory activity against SO-induced hemolysis [57]. Matsumura *et al.* 2020 conducted an *in vitro* analysis and revealed that anti-perfringolysin-O monoclonal antibody cross-reactive with SO sufficiently prevented human neutrophils from being killed by *S. pyogenes* clinical isolates [58]. Vita *et al.* 2020 reported on the *in vitro* and *ex vivo* capability of Human Serum Albumin to neutralize the cytotoxic and hemolytic effects of SO [59]. Aikawa *et al.* 2021 succeeded in obtaining a single-chain variable fragment (scFv) SLO-I4 capable of recognizing SLO, which significantly inhibited GAS-induced cell lytic activity in erythrocytes, macrophages, and epithelial cells [60]. Nozawa *et al.* 2021 found that invading GAS disrupts the Golgi complex in host cells through SO and Nga and show that GAS-induced Golgi fragmentation requires bacterial invasion into host cells [61]. Kanbayashi *et al.* 1972 looked at hemolysis of rabbit erythrocytes by SO [62]. The hemolytic rates observed at the constant rate phase were proportional to square of SO concentration and decreased as the initial concentration of erythrocyte became higher [62]. Shany *et al.* 1973 found the hemolytic activity of crude SO was completely inhibited by anti-tetanolysin [63]. Thelestam *et al.* 1980 show that membrane-damaging properties on human diploid embryonic lung fibroblasts of SO are consistent with the suggested mechanism for hemolysis involving one fixation site and one lytic site of this cytolysin [64]. Results by Tapsall *et al.* 1984 suggest that synergistic hemolysis seen in the CAMP reaction system with group A streptococci is due to the action of those small amounts of SO which remain unoxidized and thus have a capacity to lyse the fragile beta-lysin-treated sheep erythrocytes [65]. Engel *et al.* 1995 demonstrated that SO evoked permeability defects dose dependently in resected round window membrane [52]. Murai *et al.* 1996 found that culture supernatants of SPE-stimulated lymphocytes, when administered into rabbits three hours before or together with endotoxin, potentiate a variety of endotoxin-induced pathophysiological changes and even lethal shock [66]. Timmer *et al.* 2009 found that SO promotes GAS immune evasion by accelerated macrophage apoptosis [67]. Bastiat-Sempe *et al.* 2014 showed, using isogenic mutants, that the GAS pore-forming toxin SO and its cotoxin NAD-glycohydrolase (NADase) mediate GAS intracellular survival and cytotoxicity for macrophages [68]. Velarde *et al.* 2017 characterize the binding interaction between NADase and SO and reports that the expression of each toxin is crucial for maximal expression and stability of the other [69]. By this mechanism, the presence of both toxins increases toxicity to keratinocytes. Sogawa *et al.* 2018 showed that hydroxytyrosol inhibited SO-induced hemolytic activity [57]. Cheng *et al.* 2019 show that SO of GAS is identified as a crucial inducer of Reactive Oxygen Species for β1 integrin-mediated LAP (LC3-associated phagocytosis) induction [70]. Hancz *et al.* 2019 use a macrophage infection model to show that SO specifically induces ubiquitination and degradation of pro-IL-1β [71]. Aikawa *et al.* 2021 succeeded in obtaining a single-chain variable fragment (scFv) SO-I4 capable of recognizing SO, which significantly inhibited GAS-induced cell lytic activity in erythrocytes [60]. Murase *et al.* 2021 showed invasive GAS extra-cellular vesicles showed SO-dependent cytotoxic activity and the induction of cytokine expression [72]. Nozawa *et al.* 2021 show that GAS-induced Golgi fragmentation requires bacterial invasion into host cells, SO pore formation activity, and Nga NADase activity [61]. |
| Streptolysin S (SS) | Ofek *et al.* 1972 found all the forms of SS used, at 100 hemolytic units, killed mouse leukocyte monolayers [73]. Ofek *et al.* 1990 found cytolytic activity towards tissue culture cells and mouse peritoneal macrophages by SS was greater in mixtures containing organisms capable of adhering to target cells compared with mixtures with nonadherent bacteria [74]. Flaherty *et al.* 2018 found that Interleukin-1beta was found to be markedly upregulated in the presence of SS, and further investigation revealed that this cytokine (in an SS-dependent manner) contributes to cytotoxicity in human keratinocytes during infection [75]. Okazaki *et al.* 1971 discovered results that suggest that SS induces lysis by modifying the semi permeable character (impermeability to cations) of cell membranes [76]. Thelestam *et al.* 1975 evaluated human diploid fibroblast membrane damage through release of an acid from the cytoplasm of prelabelled fibroblast cells - SS at low concentrations induced a significant release of the label [77]. Hryniewicz *et al.* 1981 found that SS in sub-cytotoxic concentrations may alter lymphocyte membrane properties [78]. Carr *et al.* 2001 showed that although SS could bind to erythrocytes below 17 °C; however, lysis could only occur at temperatures >23 °C [79]. Flaherty *et al.* 2015 identified key SS-dependent host responses, including the initiation of specific programmed cell death and inflammatory cascades [80]. Higashi *et al.* 2016 show, using high-resolution live cell imaging, that SS induces a dramatic osmotic change in red blood cells, leading to cell lysis [81]. Flaherty *et al.* 2018 demonstrate that IL-1β was found to be markedly upregulated in the presence of SS, and further investigation revealed that this cytokine contributes to cytotoxicity in human keratinocytes during infection [75]. Tsao *et al.* 2019 show that SS induces mitochondrial damage and macrophage death through inhibiting degradation of glycogen synthase kinase-3β [82]. |
| Streptococcal pyrogenic exotoxin (SPE), Superantigen | Treatment of peripheral blood mononuclear cells with streptococcal pyrogenic toxins A and C was found to significantly upregulates expression of cutaneous lymphocyte-associated antigen, by Leung *et al.* 1995 [83]. This supports the idea of superantigens selectively driving localisation of activated T cells that initiate cytokine-driven inflammation characteristic of psoriasis as well as defective differentiation of keratinocytes that results in the formation of psoriatic plaque. Smoot *et al.* 2002 found that SPEs L and M were pyrogenic and mitogenic for rabbit splenocytes and human peripheral blood mononuclear cells in picogram amounts, *in vitro [84].* SpeL preferentially expanded human T cells expressing various V-beta receptors, with SpeM doing the same but with only one receptor, indicating superantigenicity. Fleischer *et al.* 1996 found similar with SPE A and C [85]. SPE A induced TNF production in a dose-dependent manner, in human peripheral blood monocytes in a study by Fast *et al.* 1989 [86]. After human monocyte stimulation with SPE A, Hackett and Stevens, 1992 found it a potent inducer of TNF-alpha, and synergistically regulated Interleukin-1beta production [51]. Leonard *et al.* 1992 found immune-lethality was observed when human lymphocytes were treated with both SPE and LPS [87]. Simultaneous addition of both was required since sequential addition did not exhibit the same effect. Mollick *et al.* 1992 showed that a streptococcal antigen stimulated human T cells that expressed various beta families [88]. Hackett and Stevens 1993 showed that SPE A is a potent inducer of Total TNF in peripheral blood mononuclear cells [89]. Hensler *et al.* 1993 showed that SPE A affects the signal transduction pathways of human polymorphonuclear granulocytes, which results in immunomodulatory functions [90]. In a study by Bussing *et al.* 1995, cultivation of peripheral blood mononuclear cells in the presence of SPE A and C resulted in significant induction of sister chromatid exchange (SCE)-inducing DNA lesions [91]. Kline *et al.* 1996 found that SPEs A and C were able to mitogenically stimulate human peripheral blood mononuclear cells, and exhibit affinity for class II MHC molecules [92]. Imanishi *et al.* 1995 showed that TNF production by human T-cells could be stimulated through SPE A [93]. Murai *et al.* 1996 found that SPE-induced lymphokines mediate the potentiating effect of SPE on the lethal endotoxin shock through enhancing endotoxin reactivity of macrophages which play the central role in mediating endotoxin toxicity [66]. Rink *et al.* 1996 found SPE B induced lower cytokine release in human peripheral blood mononuclear cells than C, but similar amounts of IL-6 and IL-10 [94]. Christ *et al.* 1997 showed that SPEs A and C individually, in the presence of IFN-gamma only, were able to trigger production of inducible nitric oxide synthase [95]. Muller-Alouf *et al.* 1997 demonstrated the ability of SPE A to stimulate release of hematopoietic and immunoregulatory cytokines by human peripheral blood mononuclear cells [96]. SPE F caused permeabilization of rat lung blood vessels, as shown by Matsumoto *et al.* 1999 [97]. Watanabe *et al.* 2002 showed secreted SPE and Streptococcal Cysteine Protease (SCP) induced histamine release and degranulation of the human mast cell line HMC-1 culture [98]. SPE A caused lethal shock induced in mice transgenic for human leukocyte antigen and human CD4 receptors, accompanied by massive cytokine production, in a study by Welcher *et al.* 2002 [99]. Hsu *et al.* 2008 suggest SPE B causes immunosuppression by cleaving human S-adenosylhomocysteine hydrolase [100]. Davies *et al.* 2019 found a marked B cell apoptosis and abrogation of total Ig production occurrence in the presence of SPE A [101]. Group A SPE types A, B, and C and staphylococcal pyrogenic exotoxin were shown to be potent nonspecific T-lymphocyte mitogens in a study by Schlievert *et al.* 1979 [102]. Knoll *et al.* 1982 showed SPEs A and C have strong evidence of mitogenic activity [103]. Concentrates of SPEs A, B and C had mitogenic and pyrogenic activity shown in a study by Gerlach *et al.* 1994 [104]. Norrby-Teglund *et al.* 1994 showed that SpeF could preferentially activate T cells bearing V beta 2, 4, 8, 15, and 19, as determined by quantitative PCR [105]. Bussing *et al.* 1995 showed that cultivation of PBMCs in the presence of SPEs A and C resulted in a significant induction of sister chromatid exchange (SCE)-inducing DNA lesions [91]. Results by Dobashi *et al.* 1999 suggest that SPE A Kupffer cells produce IL-12 and other monokines, while also non-specifically activating both NK cells and NK1(+) T cells to produce IFN-gamma [106]. Kuo *et al.* 1999 show that SPE B possesses the ability to induce apoptosis in monocytic cells [107]. Results from a study by Matsumoto *et al.* 1999 suggest that streptococcal SPE F is a major cause of permeabilization of lung blood vessels and sufficient for the pathogenesis of ARDS [97]. Nemoto *et al.* 1996 discovered that SPE M (unlike the other SPE’s) exhibited potent mitogenic activity on human T cells and V beta 21+ T cells were selectively expanded [108]. Ohara-Nemoto *et al.* 1996 show evidence of expression of T-cell receptor V beta 2 and type 1 helper T-cell-related cytokine mRNA in SPE C-activated human PBMCs [109]. When looking at VBeta profiles in rabbit lymphocytes *in vitro,* Kamezawa *et al.* found that Vbeta 8 subfamily elicitation was unique to SME Z, while the Vbeta 2 and 6 subfamilies were found to be common among lymphocytes stimulated with SPEs A, B, C, or SMEZ [12]. Szczepanska *et al.* 1999 demonstrated that Th1-type cytokine mRNA in rheumatoid arthritis mononuclear cells induced by SPE A [110]. In a study by Gerlach *et al.* 2001, mitogenic activity could be only neutralized by antibodies against the basic streptococcal superantigens SPEC or SPEX (SMEZ3) [111]. Proft *et al.* 2001 found that recombinant forms of SPE I and SPE J were mitogenic for human PBL [112]. Proft *et al.* 2003 reported that Recombinant SPE L (rSPE L) and rSPE M were highly mitogenic for human peripheral blood lymphocytes [113]. Chiang-Ni *et al.* 2006 reported that polymorphonuclear (PMN) cells' mitochondria showed decreasing dehydrogenase activity and loss of membrane potential after r-SpeB treatment, showing indication of mitochondria damage to PMN cells [114]. Nilsson *et al.* 2006 demonstrate that SO activates human PMNs) by perforating these cells; PMNs then secrete heparin-binding protein, a potent inducer of vascular leakage, and neutrophil-borne proteins, including LL-37, alpha-defensins, and elastase [115]. Luo *et al.* 2010 show authors show that anti-SPE B antibodies exhibited characteristics of autoantibodies, which cross-react with endothelial cells [116]. Giesbrecht *et al.* 2019 show that monocytes activated by SPE A mediate apoptosis of CD4+Foxp3− T effector cells through PD-L1 and kynurenine [117]. |
| M protein | Beachey *et al.* 1971 found that highly purified M protein preparations have potent in vitro cytotoxic effects upon platelets and polymorphonuclear leukocytes [118]. Psoriasis may be induced and exacerbated by M-protein specific Th_1_-type cells that cross react with human epidermal keratin, in a study by Valdimarsson *et al.* 1997 [119]. Herwald *et al.* 2004 showed that M protein complexes with fibrinogen, which by binding to beta-2 integrins of neutrophils, activate these cells [120]. Neutrophils then release heparin binding protein, an inflammatory mediator inducing vascular leakage. Pahlman *et al.* 2008 showed Soluble M1 protein triggers T cell proliferation and release of Th1 type cytokines in peripheral blood mononuclear cells [121]. Sigurdardottir *et al.* 2010 demonstrated M1 protein causes vascular nitric oxide production leading to hyporesponsiveness to vasosuppressors via mechanisms involving toll-like receptors [122]. Valderrama *et al.* 2017 found M protein activates the NLRP3 inflammasome *in vitro*: specifically by triggering programmed cell death in macrophages [123]. Beachey and Stollerman *et al.* 1971 found that that highly purified M protein preparations have potent in vitro cytotoxic effects upon platelets and polymorphonuclear leukocytes [118]. Kotb *et al.* 1993 found that The ability of pep M5 to stimulate interleukin-1 (IL-1) and tumor necrosis factor alpha (TNF-alpha) production by a T-cell-depleted, monocyte- and B-cell-enriched cell population was dependent on the presence of T cells, and the requirement for T cells could be met by addition of exogenous gamma interferon (IFN-gamma) [124]. |
| Polysaccharide cell wall extracts or whole | Smialowicz *et al.* 1977 showed that Fisher macrophages activated with group A cell walls were cytotoxic for L-cells [25]. Lipoteichoic acid (LTA) was able to stimulate cell division or kill tissue culture cells derived from human heart, in a study by Simpson *et al.* 1982 [125]. In a further study by Leon *et al.* 1983, LTA, in high concentrations was able to cause cell death of mouse fibroblast layers in tissue culture in the absence of serum [126]. Furthermore, in a study by Tomlinson *et al.* 1983, cultured mouse glomeruli were examined at cellular and subcellular levels post-LTA exposure [127]. Changes were characterised by initial reduction in outgrowth of cells, some cellular granulation and later, destruction of the confluent monolayer. Complete glomerular destruction resulted after two weeks. Muller-Alouf *et al.* 1994 found lipopolysaccharides (LPS) were able to stimulate cytokines from human peripheral blood mononuclear cells [128]. Ganguly *et al.* 1985 report the novel finding that tyrosine protein kinase activity is increased in human fibroblasts treated with LTA [129]. Data by Leon *et al.* 1985 suggests that partial inhibition of prolyl hydroxylase activity is directly related to the synthesis of defective collagen by wet fibroblast monolayers exposed to minute amounts of group A, type 12 streptococcal LTA [130]. Weinreb *et al.* 1986 show that the ability to render host cells susceptible to lysis by autologous complement is a general property of LTA [131]. In a study by Levy *et al.* 1990, LTA harvested from *S. pyogenes* caused direct activation of the respiratory burst in human peripheral blood monocytes [132]. Keller *et al.* 1992 found that LTAs from *S. pyogenes* were able to affect bone marrow-derived phagocyte parameters measured such as reductive capacity, secretion of tumor necrosis factor and nitrite, and tumoricidal activity [133]. LPS and PG-PS was able to cause TNF release from Kupffer cells in a study by Lichtman *et al.* 1994 [134]. Kita *et al.* 1999 used rat ears to administer LTA to and found it induced production of proinflammatory cytokines, and rat chemokines albeit differently in middle ear lavages, and in the middle ear and nasal mucosa [41]. Murr *et al.* 1997 found that in PBMCs, LPS was able to stimulate tryptophan degradation in humans via the induction of interferon-gamma production [135]. Okamato *et al.* 2000 show findings that clearly indicated that OK-PSA, an LTA-related molecule, is a main effective component of OK-432, and is a potent inducer of Th1-type cytokines by T cell and natural killer (NK) cell activation mediated by monocytes-derived IL-18 [136]. Maudsdotter *et al.* 2011 demonstrate that lactic acid reduces epithelial cell damage caused by GAS by degrading both secreted and cell-bound LTA [137]. |
| Erythrogenic toxins (ET) | Streptococcal erythrogenic toxins A, B and C and Streptococcal-derived Mitogen-BX were able to stimulate neopterin in human peripheral blood mononuclear cell lines via the induction of huge amounts of interferon-gamma, shown by Murr *et al.* 1997 [135]. Buslau *et al.* 1993 showed Significant T cell proliferation in response to minute amounts of ETA, in human epidermal cells [138]. |
| NADase | Velarde *et al.* 2017 found that Expression of NADase by streptococcus either enzymatically active or inactive, augments SLO-mediated toxicity for keratinocytes [69]. Michos *et al.* 2006 showed that exposure of human keratinocytes to wild-type GAS, but not to a NADase-deficient mutant strain, resulted in profound depletion of cellular NADⴙ and ATP [139]. Furthermore, expression of recombinant GAS NADase in yeast, in the absence of SLO, induced growth arrest, depletion of NADⴙ and ATP, and cell death [139]. Sharma *et al.* 2016 showed that exposure of human oropharyngeal keratinocytes to LFnNADase in the presence of protective antigen resulted in cytosolic delivery of NADase activity, inhibition of protein synthesis, and cell death [140]. |
| Regulatory elements | Chaussee *et al.* 2002, identified Rgg - a regulator (via additional regulatory networks in promotor regions in genes) of a toxic exoprotein, cysteine protease B (SPE B) [141]. Eraso *et al.* found that a one nucleotide indel in an intergenic homopolymeric tract located between genes *Spy1336/R28* and *Spy1337* results in upregulation of NADase cytotoxin; slo encoding cytolytic protein sin O, as well as the sag operon encoding streptolysin S, and amm28 encoding M protein [142]. Zhang *et al.* 2012 showed inhibition of Rho-kinase signalling significantly reduced M1 protein-provoked neutrophil accumulation and edema formation in the lung [143]. Through suppression of regulatory regions ropB, sagA and dltA by botulin, Viszwapriya *et al.* 2016 found production of SPE B and hemolysis changed [144]. Theodore *et al.* 1981 found that lipoteichoic acid from streptococci and activated membrane-bound precursor streptolysin S and induced the formation of extracellular streptolysin S [145]. Federle *et al.* 1999 found that nonpolar insertion in regulatory gene CovR caused increased transcription of sagA (SLS) and speMF (mitogenic factor) [146]. Heath *et al.* 1999 showed that csrR mutants have enhanced transcription of sagA, a gene associated with streptolysin S and speB, the gene encoding SPE B [147]. The mutants also express substantially higher SS activity and SPE B antigen in late-exponential-phase cultures. Nizet *et al.* 2000 found that gene products of the GAS sag operon are both necessary and sufficient for SS production [148]. Engleberg *et al.* 2001 show that CsrS/CsrR is a 2-component system in Streptococcus pyogenes that negatively regulates hyaluronic capsule and several exotoxins [149]. In a study by Lyon *et al.* 2001, analyses of luxs- mutants revealed the aberrant expression of several virulence properties that are regulated in response to growth phase, including enhanced haemolytic activity, and a dramatic reduction in the expression of secreted proteolytic activity [150]. Dmitriev *et al.* 2006 showed that inactivation of rgg disrupted transcripts encoding virulence factors involved in cytolysin-mediated translocation of NAD-glycohydrolase, including the immunity factor IFS and the cytolysin SO [151]. Ma *et al.* 2009 identified a gene unique to *S. pyogenes*, called vfr, that negatively regulates SPE B [152]. Trevino *et al.* 2009 found that CovS simultaneously activates and inhibits the CovR-mediated repression of distinct subsets of Group A streptococcus virulence factor-encoding genes such as SPE B [153]. Liang *et al.* 2013 show that CovRS is involved in the upregulation of SPE B [154]. Bao *et al.* 2014 identified the covS sensor in M23ND strain as a pseudogene, resulting in the attenuation of speB function and increased expression of the genes for the chromosomal virulence factors [155]. Makthal *et al.* 2016 found that RopB of GAS controls the expression of several major virulence factors including secreted protease SPE B [156]. Port *et al.* 2017 conducted a study in which SpxA1(-) attenuation was associated with reduced expression of several toxins, including the SPE B [157]. Bernard *et al.* 2018 demonstrate that selected virulence factor genes with decreased transcript levels in the isogenic ΔrocA strain include M28_Spy0109 (encoding pilin protein), the sag operon (carrying streptolysin S biosynthesis genes), ska (encoding streptokinase [SKA]) and speB (encoding streptococcal cysteine protease B) [158]. Brouwer *et al.* 2018 identified the endopeptidase PepO as a novel growth phase-dependent regulator of SPE B in the invasive GAS M1 serotype strain 5448 [159]. DebRoy *et al.* 2021 found that CcpA affects transcript levels of GAS virulence factors SS, PrtS (IL-8 degrading proteinase) [160], and SPE B. Faozia *et al.* 2021 showed that deletion of the c-di-AMP synthase gene, dacA, results in pleiotropic effects including reduced expression of the secreted protease SPE B [161]. Wu *et al.* 2021 showed that proteins acting on detoxification of oxidative stress and induction of host cell death, such as peroxiredoxin (AhpC), NADH oxidase (NOX), superoxide dismutase (SodA) and streptolysin O (SLO) were reduced in the absence of PrsA [162]. |
| Other secretory proteins | Terao *et al.* 2008 show that component degrades C3b, the purpose of which is do opsonize the pathogen to facilitate phagocytosis [163]. |
| Unverified toxin | Schwab *et al.* 1956 report a new intracellular lysin via its action on various red blood cells, which is readily distinguished from SO and SS [164]. Marchlewicz *et al.* 1980 found a group B streptococcal hemolysin that appears to be similar to, but distinct from, streptolysin S, in its function [165]. Streptococcal mitogenic exotoxin Z (SMEZ), in a study by Muller-Alouf *et al.* 2001, provoked expansion of human lymphocytes expressing the Vbeta 2, 4, 7 and 8 motifs of T-cell receptor [166]. Coye *et al.* 2004 showed that recombinant SpyA was able to hydrolyse beta-NAD(+), and this activity was dependent on a glutamate at position 187 and expression of spyA in HeLa cells resulted in loss of actin microfilaments [167]. |

| **Table S3. Components of *Streptococcus pyogenes* found to demonstrate allergenic effects *in vivo* in humans or animals, from literature reviews performed on or before 31^st^ March 2022** | |
| --- | --- |
| Allergenic component | Studies demonstrating effects (*in vivo*) |
| Lipoteichoic acid | Fiedel and Jackson, 1976 administered Teichoic acids (TAs) as injections through various routes, in mice and rabbits [168]. With soluble TA, they found no evidence of antibody response. However, in rabbits, with double emulsion of Freund incomplete adjuvant with mBSA, antibody to the TA was detectable in rabbits. After secondary injection – rabbits underwent anaphylactoid response [168]. |
| Polysaccharide cell wall extracts or whole | In a study by Van der Broek, 1988, cell-wall primed as well as naïve mice, arthritis was induced in a dose dependent manner [169]. Authors proposed that a cross-reactive antibody response against cell-wall fragments could be elicited and suggest a mechanism for pathology of chronic arthritis. Van den Bruggen *et al.*, 1991 showed that in streptococcal cell wall induced arthritis, antigen-specific T cells have been demonstrated as crucial for chronic disease - susceptibility mediated by immune cells [170]. Esser *et al.* 1985 showed that joint inflammation initially induced by intraarticular injection of an aqueous suspension of peptidoglycan-polysaccharide (PG-PS) fragments isolated from *S. pyogenes* was reactivated by systemic injection of a normally subarthropathic dose of homologous or heterologous cell wall polymers [171]. |

1. Zhu L, Olsen RJ, Lee JD, Porter AR, DeLeo FR, Musser JM: **Contribution of Secreted NADase and Streptolysin O to the Pathogenesis of Epidemic Serotype M1 Streptococcus pyogenes Infections.** *Am J Pathol* 2017, **187:**605-613.

2. Kaplan EL, Wannamaker LW: **Suppression of the antistreptolysin O response by cholesterol and by lipid extracts of rabbit skin.** *J Exp Med* 1976, **144:**754-767.

3. Shanley TP, Schrier D, Kapur V, Kehoe M, Musser JM, Ward PA: **Streptococcal cysteine protease augments lung injury induced by products of group A streptococci.** *Infect Immun* 1996, **64:**870-877.

4. Bryant AE, Bayer CR, Chen RY, Guth PH, Wallace RJ, Stevens DL: **Vascular dysfunction and ischemic destruction of tissue in Streptococcus pyogenes infection: the role of streptolysin O-induced platelet/neutrophil complexes.** *J Infect Dis* 2005, **192:**1014-1022.

5. Brosnahan AJ, Mantz MJ, Squier CA, Peterson ML, Schlievert PM: **Cytolysins augment superantigen penetration of stratified mucosa.** *J Immunol* 2009, **182:**2364-2373.

6. Chiarot E, Faralla C, Chiappini N, Tuscano G, Falugi F, Gambellini G, Taddei A, Capo S, Cartocci E, Veggi D, et al: **Targeted amino acid substitutions impair streptolysin O toxicity and group A Streptococcus virulence.** *mBio* 2013, **4:**e00387-00312.

7. Abe Y, Nakano S, Aita K, Sagishima M: **Erythrogenic toxin-induced arteritis in a rabbit ear model. Comparison with Arthus reaction angiitis.** *Adv Exp Med Biol* 1997, **418:**805-807.

8. Limbago B, Penumalli V, Weinrick B, Scott JR: **Role of streptolysin O in a mouse model of invasive group A streptococcal disease.** *Infect Immun* 2000, **68:**6384-6390.

9. Fontaine MC, Lee JJ, Kehoe MA: **Combined contributions of streptolysin O and streptolysin S to virulence of serotype M5 Streptococcus pyogenes strain Manfredo.** *Infect Immun* 2003, **71:**3857-3865.

10. Smoot JC, Barbian KD, Van Gompel JJ, Smoot LM, Chaussee MS, Sylva GL, Sturdevant DE, Ricklefs SM, Porcella SF, Parkins LD, et al: **Genome sequence and comparative microarray analysis of serotype M18 group A Streptococcus strains associated with acute rheumatic fever outbreaks.** *Proc Natl Acad Sci U S A* 2002, **99:**4668-4673.

11. Murai T, Ogawa Y, Kawasaki H, Kanoh S: **Physiology of the potentiation of lethal endotoxin shock by streptococcal pyrogenic exotoxin in rabbits.** *Infect Immun* 1987, **55:**2456-2460.

12. Kamezawa Y, Nakahara T: **Purification and characterization of streptococcal erythrogenic toxin type A produced by Streptococcus pyogenes strain NY-5 cultured in the synthetic medium NCTC-135. Comparison with the dialyzed medium (TP medium)-derived toxin.** *Microbiol Immunol* 1989, **33:**183-194.

13. Lee PK, Schlievert PM: **Quantification and toxicity of group A streptococcal pyrogenic exotoxins in an animal model of toxic shock syndrome-like illness.** *J Clin Microbiol* 1989, **27:**1890-1892.

14. Arad G, Hillman D, Levy R, Kaempfer R: **Superantigen antagonist blocks Th1 cytokine gene induction and lethal shock.** *J Leukoc Biol* 2001, **69:**921-927.

15. Saouda M, Wu W, Conran P, Boyle MD: **Streptococcal pyrogenic exotoxin B enhances tissue damage initiated by other Streptococcus pyogenes products.** *J Infect Dis* 2001, **184:**723-731.

16. Hanna EE, Watson DW: **Host-parasite relationships among group A streptococci. IV. Suppression of antibody response by streptococcal pyrogenic exotoxin.** *J Bacteriol* 1968, **95:**14-21.

17. Hanna EE, Watson DW: **HOST-PARASITE RELATIONSHIPS AMONG GROUP A STREPTOCOCCI. 3. DEPRESSION OF RETICULOENDOTHELIAL FUNCTION BY STREPTOCOCCAL PYROGENIC EXOTOXINS.** *J Bacteriol* 1965, **89:**154-158.

18. Kamezawa Y, Nakahara T, Abe Y, Kato I: **Increased vascular permeability, erythema, and leukocyte emigration induced in rabbit skin by streptococcal erythrogenic toxin type A.** *FEMS Microbiol Lett* 1990, **56:**159-162.

19. Akiyama T, Shimanuki K, Yashiro K: **Possible role of Streptococcus pyogenes in mucocutaneous lymph node syndrome. XV. Potential utility of streptococcal pyrogenic exotoxin toxoid for the prophylaxis and treatment of MCLS.** *Acta Paediatr Jpn* 1992, **34:**516-524.

20. Kuo CF, Wu JJ, Lin KY, Tsai PJ, Lee SC, Jin YT, Lei HY, Lin YS: **Role of streptococcal pyrogenic exotoxin B in the mouse model of group A streptococcal infection.** *Infect Immun* 1998, **66:**3931-3935.

21. Lukomski S, Montgomery CA, Rurangirwa J, Geske RS, Barrish JP, Adams GJ, Musser JM: **Extracellular cysteine protease produced by Streptococcus pyogenes participates in the pathogenesis of invasive skin infection and dissemination in mice.** *Infect Immun* 1999, **67:**1779-1788.

22. Schmidt KH, Wiesner J, Gerlach D, Reichardt W, Ozegowski JH, Köhler W: **Susceptibility of chicken embryos to group A streptococci: correlation with fibrinogen binding.** *FEMS Immunol Med Microbiol* 1993, **7:**231-240.

23. Soehnlein O, Oehmcke S, Ma X, Rothfuchs AG, Frithiof R, van Rooijen N, Mörgelin M, Herwald H, Lindbom L: **Neutrophil degranulation mediates severe lung damage triggered by streptococcal M1 protein.** *Eur Respir J* 2008, **32:**405-412.

24. Zhang S, Rahman M, Zhang S, Jeppsson B, Herwald H, Thorlacius H: **Streptococcal m1 protein triggers farnesyltransferase-dependent formation of CXC chemokines in alveolar macrophages and neutrophil infiltration of the lungs.** *Infect Immun* 2012, **80:**3952-3959.

25. Smialowicz RJ, Schwab JH: **Cytotoxicity of rat macrophages activated by persistent or biodegradable bacterial cell walls.** *Infect Immun* 1977, **17:**599-606.

26. Schwab JH, Cromartie WJ, Roberson BS: **Identification of a toxic cellular component of group A streptococci as a complex of group-specific C polysaccharide and a protein.** *J Exp Med* 1959, **109:**43-54.

27. Roberson BS, Schwab JH, Cromartie WJ: **Relation of particle size of C polysaccharide complexes of group A streptococci to toxic effects on connective tissue.** *J Exp Med* 1960, **112:**751-764.

28. Cromartie WJ, Craddock JG, Schwab JH, Anderle SK, Yang CH: **Arthritis in rats after systemic injection of streptococcal cells or cell walls.** *J Exp Med* 1977, **146:**1585-1602.

29. Haraoui B, Wilder RL, Allen JB, Sporn MB, Helfgott RK, Brinckerhoff CE: **Dose-dependent suppression by the synthetic retinoid, 4-hydroxyphenyl retinamide, of streptococcal cell wall-induced arthritis in rats.** *Int J Immunopharmacol* 1985, **7:**903-916.

30. Woolverton CJ, White JJ, Jr., Sartor RB: **Eicosanoid regulation of acute intestinal vascular permeability induced by intravenous peptidoglycan-polysaccharide polymers.** *Agents Actions* 1989, **26:**301-309.

31. Müller-Alouf H, Alouf JE, Gerlach D, Fitting C, Cavaillon JM: **Cytokine production by murine cells activated by erythrogenic toxin type A superantigen of Streptococcus pyogenes.** *Immunobiology* 1992, **186:**435-448.

32. Fuseler JW, Conner EM, Davis JM, Wolf RE, Grisham MB: **Cytokine and nitric oxide production in the acute phase of bacterial cell wall-induced arthritis.** *Inflammation* 1997, **21:**113-131.

33. Yokoi K, Mukaida N, Harada A, Watanabe Y, Matsushima K: **Prevention of endotoxemia-induced acute respiratory distress syndrome-like lung injury in rabbits by a monoclonal antibody to IL-8.** *Lab Invest* 1997, **76:**375-384.

34. Roberson BS, Schwab JH: **Endotoxic properties associated with cell walls of group A streptococci.** *J Infect Dis* 1961, **108:**25-34.

35. Schwab JH: **Analysis of the experimental lesion of connective tissue produced by a complex of C polysaccharide from group A streptococci. I. In vivo reaction between tissue and toxin.** *J Exp Med* 1962, **116:**17-28.

36. Schwab JH, Allen JB, Anderle SK, Dalldorf F, Eisenberg R, Cromartie WJ: **Relationship of complement to experimental arthritis induced in rats with streptococcal cell walls.** *Immunology* 1982, **46:**83-88.

37. Fox A, Hammer ME, Lill P, Burch TG, Burrish G: **Experimental uveitis. Elicited by peptidoglycan-polysaccharide complexes, lipopolysaccharide, and muramyl dipeptide.** *Arch Ophthalmol* 1984, **102:**1063-1067.

38. Hamada S, Yamamoto T, Koga T, McGhee JR, Michalek SM, Yamamoto S: **Chemical properties and immunobiological activities of streptococcal lipoteichoic acids.** *Zentralbl Bakteriol Mikrobiol Hyg A* 1985, **259:**228-243.

39. Wells A, Pararajasegaram G, Baldwin M, Yang CH, Hammer M, Fox A: **Uveitis and arthritis induced by systemic injection of streptococcal cell walls.** *Invest Ophthalmol Vis Sci* 1986, **27:**921-925.

40. Leon O, Panos C: **An electron microscope study of kidney basement membrane changes in the mouse by lipoteichoic acid from Streptococcus pyogenes.** *Can J Microbiol* 1987, **33:**709-717.

41. Kita H, Himi T: **Cytokine and chemokine induction using cell wall component and toxin derived from gram-positive bacteria in the rat middle ear.** *Acta Otolaryngol* 1999, **119:**446-452.

42. Middelveld RJ, Alving K: **Synergistic septicemic action of the gram-positive bacterial cell wall components peptidoglycan and lipoteichoic acid in the pig in vivo.** *Shock* 2000, **13:**297-306.

43. Cho KH, Kang SO: **Streptococcus pyogenes c-di-AMP phosphodiesterase, GdpP, influences SpeB processing and virulence.** *PLoS One* 2013, **8:**e69425.

44. Nandakumar KS, Collin M, Olsén A, Nimmerjahn F, Blom AM, Ravetch JV, Holmdahl R: **Endoglycosidase treatment abrogates IgG arthritogenicity: importance of IgG glycosylation in arthritis.** *Eur J Immunol* 2007, **37:**2973-2982.

45. Honda-Ogawa M, Ogawa T, Terao Y, Sumitomo T, Nakata M, Ikebe K, Maeda Y, Kawabata S: **Cysteine proteinase from Streptococcus pyogenes enables evasion of innate immunity via degradation of complement factors.** *J Biol Chem* 2013, **288:**15854-15864.

46. Yoshizawa N, Oshima S, Takeuchi A, Kondo S, Oda T, Shimizu J, Nishiyama J, Ishida A, Nakabayashi I, Tazawa K, Sakurai Y: **Experimental acute glomerulonephritis induced in the rabbit with a specific streptococcal antigen.** *Clin Exp Immunol* 1997, **107:**61-67.

47. Hurley BP, Pirzai W, Eaton AD, Harper M, Roper J, Zimmermann C, Ladics GS, Layton RJ, Delaney B: **An experimental platform using human intestinal epithelial cell lines to differentiate between hazardous and non-hazardous proteins.** *Food Chem Toxicol* 2016, **92:**75-87.

48. Van Epps DE, Andersen BR: **Streptolysin O inhibition of neutrophil chemotaxis and mobility: nonimmune phenomenon with species specificity.** *Infect Immun* 1974, **9:**27-33.

49. Miller H, Song W: **Use of Streptolysin O-Induced Membrane Damage as a Method of Studying the Function of Lipid Rafts During B Cell Activation.** *Methods Mol Biol* 2018, **1707:**235-241.

50. Bhakdi S, Tranum-Jensen J, Sziegoleit A: **Mechanism of membrane damage by streptolysin-O.** *Infect Immun* 1985, **47:**52-60.

51. Hackett SP, Stevens DL: **Streptococcal toxic shock syndrome: synthesis of tumor necrosis factor and interleukin-1 by monocytes stimulated with pyrogenic exotoxin A and streptolysin O.** *J Infect Dis* 1992, **165:**879-885.

52. Engel F, Blatz R, Kellner J, Palmer M, Weller U, Bhadki S: **Breakdown of the round window membrane permeability barrier evoked by streptolysin O: possible etiologic role in development of sensorineural hearing loss in acute otitis media.** *Infect Immun* 1995, **63:**1305-1310.

53. Ginsburg I, Sadovnic M: **Gamma globulin, Evan's blue, aprotinin A PLA2 inhibitor, tetracycline and antioxidants protect epithelial cells against damage induced by synergism among streptococcal hemolysins, oxidants and proteinases: relation to the prevention of post-streptococcal sequelae and septic shock.** *FEMS Immunol Med Microbiol* 1998, **22:**247-256.

54. Sierig G, Cywes C, Wessels MR, Ashbaugh CD: **Cytotoxic effects of streptolysin o and streptolysin s enhance the virulence of poorly encapsulated group a streptococci.** *Infect Immun* 2003, **71:**446-455.

55. Chandrasekaran S, Caparon MG: **The NADase-Negative Variant of the Streptococcus pyogenes Toxin NAD⁺ Glycohydrolase Induces JNK1-Mediated Programmed Cellular Necrosis.** *mBio* 2016, **7:**e02215-02215.

56. Escajadillo T, Olson J, Luk BT, Zhang L, Nizet V: **A Red Blood Cell Membrane-Camouflaged Nanoparticle Counteracts Streptolysin O-Mediated Virulence Phenotypes of Invasive Group A Streptococcus.** *Front Pharmacol* 2017, **8:**477.

57. Sogawa K, Kobayashi M, Suzuki J, Sanda A, Kodera Y, Fukuyama M: **Inhibitory Activity of Hydroxytyrosol against Streptolysin O-Induced Hemolysis.** *Biocontrol Sci* 2018, **23:**77-80.

58. Matsumura T, Nishiyama A, Aiko M, Ainai A, Ikebe T, Chiba J, Ato M, Takahashi Y: **An anti-perfringolysin O monoclonal antibody cross-reactive with streptolysin O protects against streptococcal toxic shock syndrome.** *BMC Res Notes* 2020, **13:**419.

59. Vita GM, De Simone G, Leboffe L, Montagnani F, Mariotti D, Di Bella S, Luzzati R, Gori A, Ascenzi P, di Masi A: **Human Serum Albumin Binds Streptolysin O (SLO) Toxin Produced by Group A Streptococcus and Inhibits Its Cytotoxic and Hemolytic Effects.** *Front Immunol* 2020, **11:**507092.

60. Aikawa C, Kawashima K, Fukuzaki C, Nakakido M, Murase K, Nozawa T, Tsumoto K, Nakagawa I: **Single-chain variable fragment (scFv) targeting streptolysin O controls group A Streptococcus infection.** *Biochem Biophys Res Commun* 2021, **566:**177-183.

61. Nozawa T, Iibushi J, Toh H, Minowa-Nozawa A, Murase K, Aikawa C, Nakagawa I: **Intracellular Group A Streptococcus Induces Golgi Fragmentation To Impair Host Defenses through Streptolysin O and NAD-Glycohydrolase.** *mBio* 2021, **12**.

62. Kanbayashi Y, Hotta M, Koyama J: **Kinetic study on streptolysin O.** *J Biochem* 1972, **71:**227-237.

63. Shany S, Grushoff PS, Bernheimer AW: **Physical separation of streptococcal nicotinamide adenine dinucleotide glycohydrolase from streptolysin O.** *Infect Immun* 1973, **7:**731-734.

64. Thelestam M, Möllby R: **Interaction of streptolysin O from Streptococcus pyogenes and theta-toxin from Clostridium perfringens with human fibroblasts.** *Infect Immun* 1980, **29:**863-872.

65. Tapsall JW, Phillips EA: **Streptococcus pyogenes streptolysin O as a cause of false-positive CAMP reactions.** *J Clin Microbiol* 1984, **19:**534-537.

66. Murai T, Nakagawa Y, Ogawa Y: **Potentiation of lethal endotoxin shock by streptococcal pyrogenic exotoxin in rabbits: possible relevance of hyperreactivity of macrophages to endotoxin.** *FEMS Immunol Med Microbiol* 1996, **13:**269-272.

67. Timmer AM, Timmer JC, Pence MA, Hsu LC, Ghochani M, Frey TG, Karin M, Salvesen GS, Nizet V: **Streptolysin O promotes group A Streptococcus immune evasion by accelerated macrophage apoptosis.** *J Biol Chem* 2009, **284:**862-871.

68. Bastiat-Sempe B, Love JF, Lomayesva N, Wessels MR: **Streptolysin O and NAD-glycohydrolase prevent phagolysosome acidification and promote group A Streptococcus survival in macrophages.** *mBio* 2014, **5:**e01690-01614.

69. Velarde JJ, O'Seaghdha M, Baddal B, Bastiat-Sempe B, Wessels MR: **Binding of NAD(+)-Glycohydrolase to Streptolysin O Stabilizes Both Toxins and Promotes Virulence of Group A Streptococcus.** *mBio* 2017, **8**.

70. Cheng YL, Kuo CF, Lu SL, Omori H, Wu YN, Hsieh CL, Noda T, Wu SR, Anderson R, Lin CF, et al: **Group A Streptococcus Induces LAPosomes via SLO/β1 Integrin/NOX2/ROS Pathway in Endothelial Cells That Are Ineffective in Bacterial Killing and Suppress Xenophagy.** *mBio* 2019, **10**.

71. Hancz D, Westerlund E, Valfridsson C, Aemero GM, Bastiat-Sempe B, Orning P, Lien E, Wessels MR, Persson JJ: **Streptolysin O Induces the Ubiquitination and Degradation of Pro-IL-1β.** *J Innate Immun* 2019, **11:**457-468.

72. Murase K, Aikawa C, Nozawa T, Nakatake A, Sakamoto K, Kikuchi T, Nakagawa I: **Biological Effect of Streptococcus pyogenes-Released Extracellular Vesicles on Human Monocytic Cells, Induction of Cytotoxicity, and Inflammatory Response.** *Front Cell Infect Microbiol* 2021, **11:**711144.

73. Ofek I, Bergner-Rabinowitz S, Ginsburg I: **Oxygen-stable hemolysins of group A streptococci. 8. Leukotoxic and antiphagocytic effects of streptolysins S and O.** *Infect Immun* 1972, **6:**459-464.

74. Ofek I, Zafriri D, Goldhar J, Eisenstein BI: **Inability of toxin inhibitors to neutralize enhanced toxicity caused by bacteria adherent to tissue culture cells.** *Infect Immun* 1990, **58:**3737-3742.

75. Flaherty RA, Donahue DL, Carothers KE, Ross JN, Ploplis VA, Castellino FJ, Lee SW: **Neutralization of Streptolysin S-Dependent and Independent Inflammatory Cytokine IL-1β Activity Reduces Pathology During Early Group A Streptococcal Skin Infection.** *Front Cell Infect Microbiol* 2018, **8:**211.

76. Okazaki H: **Streptolysin S' of Streptococcus pyogenes: studies on phospholipase activity.** *J Biochem* 1971, **70:**867-868.

77. Thelestam M, Möllby R: **Sensitive assay for detection of toxin-induced damage to the cytoplasmic membrane of human diploid fibroblasts.** *Infect Immun* 1975, **12:**225-232.

78. Hryniewicz W, Roszkowski W, Lipski S, Jeljaszewicz J: **Influence of streptolysin S from Streptococcus pyogenes on some functions of lymphocyte membrane.** *Toxicon* 1981, **19:**33-39.

79. Carr A, Sledjeski DD, Podbielski A, Boyle MD, Kreikemeyer B: **Similarities between complement-mediated and streptolysin S-mediated hemolysis.** *J Biol Chem* 2001, **276:**41790-41796.

80. Flaherty RA, Puricelli JM, Higashi DL, Park CJ, Lee SW: **Streptolysin S Promotes Programmed Cell Death and Enhances Inflammatory Signaling in Epithelial Keratinocytes during Group A Streptococcus Infection.** *Infect Immun* 2015, **83:**4118-4133.

81. Higashi DL, Biais N, Donahue DL, Mayfield JA, Tessier CR, Rodriguez K, Ashfeld BL, Luchetti J, Ploplis VA, Castellino FJ, Lee SW: **Activation of band 3 mediates group A Streptococcus streptolysin S-based beta-haemolysis.** *Nat Microbiol* 2016, **1:**15004.

82. Tsao N, Kuo CF, Cheng MH, Lin WC, Lin CF, Lin YS: **Streptolysin S induces mitochondrial damage and macrophage death through inhibiting degradation of glycogen synthase kinase-3β in Streptococcus pyogenes infection.** *Sci Rep* 2019, **9:**5371.

83. Leung DY, Gately M, Trumble A, Ferguson-Darnell B, Schlievert PM, Picker LJ: **Bacterial superantigens induce T cell expression of the skin-selective homing receptor, the cutaneous lymphocyte-associated antigen, via stimulation of interleukin 12 production.** *J Exp Med* 1995, **181:**747-753.

84. Smoot LM, McCormick JK, Smoot JC, Hoe NP, Strickland I, Cole RL, Barbian KD, Earhart CA, Ohlendorf DH, Veasy LG, et al: **Characterization of two novel pyrogenic toxin superantigens made by an acute rheumatic fever clone of Streptococcus pyogenes associated with multiple disease outbreaks.** *Infect Immun* 2002, **70:**7095-7104.

85. Fleischer B, Necker A, Leget C, Malissen B, Romagne F: **Reactivity of mouse T-cell hybridomas expressing human Vbeta gene segments with staphylococcal and streptococcal superantigens.** *Infect Immun* 1996, **64:**987-994.

86. Fast DJ, Schlievert PM, Nelson RD: **Toxic shock syndrome-associated staphylococcal and streptococcal pyrogenic toxins are potent inducers of tumor necrosis factor production.** *Infect Immun* 1989, **57:**291-294.

87. Leonard BA, Schlievert PM: **Immune cell lethality induced by streptococcal pyrogenic exotoxin A and endotoxin.** *Infect Immun* 1992, **60:**3747-3755.

88. Mollick JA, Miller GG, Musser JM, Cook RG, Rich RR: **Isolation and characterization of a novel streptococcal superantigen.** *Trans Assoc Am Physicians* 1992, **105:**110-122.

89. Hackett SP, Stevens DL: **Superantigens associated with staphylococcal and streptococcal toxic shock syndrome are potent inducers of tumor necrosis factor-beta synthesis.** *J Infect Dis* 1993, **168:**232-235.

90. Hensler T, Köller M, Geoffroy C, Alouf JE, König W: **Staphylococcus aureus toxic shock syndrome toxin 1 and Streptococcus pyogenes erythrogenic toxin A modulate inflammatory mediator release from human neutrophils.** *Infect Immun* 1993, **61:**1055-1061.

91. Büssing A, Klotz M, Suzart K, Efferth T, Gerlach D, Schnitzler N, Osieka R, Schweizer K, Kaufhold A: **Sister chromatid exchange-inducing DNA lesions and depression of activation markers on the surface of cultured peripheral blood mononuclear cells after the addition of streptococcal pyrogenic exotoxins A and C.** *Med Microbiol Immunol* 1995, **184:**87-96.

92. Kline JB, Collins CM: **Analysis of the superantigenic activity of mutant and allelic forms of streptococcal pyrogenic exotoxin A.** *Infect Immun* 1996, **64:**861-869.

93. Imanishi K, Inada K, Akatsuka H, Gu Y, Igarashi H, Uchiyama T: **Tumor necrosis factor production by human T-cells stimulated with bacterial superantigens.** *Int J Immunopharmacol* 1995, **17:**841-848.

94. Rink L, Luhm J, Koester M, Kirchner H: **Induction of a cytokine network by superantigens with parallel TH1 and TH2 stimulation.** *J Interferon Cytokine Res* 1996, **16:**41-47.

95. Christ EA, Meals E, English BK: **Streptococcal pyrogenic exotoxins A (SpeA) and C (SpeC) stimulate the production of inducible nitric oxide synthase (iNOS) protein in RAW 264.7 macrophages.** *Shock* 1997, **8:**450-453.

96. Müller-Alouf H, Capron M, Alouf JE, Geoffroy C, Gerlach D, Ozegowski JH, Fitting C, Cavaillon JM: **Cytokine profile of human peripheral blood mononucleated cells stimulated with a novel streptococcal superantigen, SPEA, SPEC and group A streptococcal cells.** *Adv Exp Med Biol* 1997, **418:**929-931.

97. Matsumoto M, Ishikawa N, Saito M, Shibayama K, Horii T, Sato K, Ohta M: **Streptococcal pyrogenic exotoxin F (SpeF) causes permeabilization of lung blood vessels.** *Infect Immun* 1999, **67:**4307-4311.

98. Watanabe Y, Todome Y, Ohkuni H, Sakurada S, Ishikawa T, Yutsudo T, Fischetti VA, Zabriskie JB: **Cysteine protease activity and histamine release from the human mast cell line HMC-1 stimulated by recombinant streptococcal pyrogenic exotoxin B/streptococcal cysteine protease.** *Infect Immun* 2002, **70:**3944-3947.

99. Welcher BC, Carra JH, DaSilva L, Hanson J, David CS, Aman MJ, Bavari S: **Lethal shock induced by streptococcal pyrogenic exotoxin A in mice transgenic for human leukocyte antigen-DQ8 and human CD4 receptors: implications for development of vaccines and therapeutics.** *J Infect Dis* 2002, **186:**501-510.

100. Hsu JF, Chuang WJ, Shiesh SC, Lin YS, Liu CC, Wang CC, Fu TF, Tsai JH, Tsai WL, Huang YJ, et al: **Streptococcal pyrogenic exotoxin B cleaves human S-adenosylhomocysteine hydrolase and induces hypermethioninemia.** *J Infect Dis* 2008, **198:**367-374.

101. Davies FJ, Olme C, Lynskey NN, Turner CE, Sriskandan S: **Streptococcal superantigen-induced expansion of human tonsil T cells leads to altered T follicular helper cell phenotype, B cell death and reduced immunoglobulin release.** *Clin Exp Immunol* 2019, **197:**83-94.

102. Schlievert PM, Schoettle DJ, Watson DW: **Nonspecific T-lymphocyte mitogenesis by pyrogenic exotoxins from group A streptococci and Staphylococcus aureus.** *Infect Immun* 1979, **25:**1075-1077.

103. Knöll H, Holm SE, Gerlach D, Köhler W: **Tissue cages for study of experimental streptococcal infection in rabbits. I. Production of erythrogenic toxins in vivo.** *Immunobiology* 1982, **162:**128-140.

104. Gerlach D, Reichardt W, Fleischer B, Schmidt KH: **Separation of mitogenic and pyrogenic activities from so-called erythrogenic toxin type B (Streptococcal proteinase).** *Zentralbl Bakteriol* 1994, **280:**507-514.

105. Norrby-Teglund A, Newton D, Kotb M, Holm SE, Norgren M: **Superantigenic properties of the group A streptococcal exotoxin SpeF (MF).** *Infect Immun* 1994, **62:**5227-5233.

106. Dobashi H, Seki S, Habu Y, Ohkawa T, Takeshita S, Hiraide H, Sekine I: **Activation of mouse liver natural killer cells and NK1.1(+) T cells by bacterial superantigen-primed Kupffer cells.** *Hepatology* 1999, **30:**430-436.

107. Kuo CF, Wu JJ, Tsai PJ, Kao FJ, Lei HY, Lin MT, Lin YS: **Streptococcal pyrogenic exotoxin B induces apoptosis and reduces phagocytic activity in U937 cells.** *Infect Immun* 1999, **67:**126-130.

108. Nemoto E, Rikiishi H, Sugawara S, Okamoto S, Tamura K, Maruyama Y, Kumagai K: **Isolation of a new superantigen with potent mitogenic activity to murine T cells from Streptococcus pyogenes.** *FEMS Immunol Med Microbiol* 1996, **15:**81-91.

109. Ohara-Nemoto Y, Kaneko M: **Expression of T-cell receptor V beta 2 and type 1 helper T-cell-related cytokine mRNA in streptococcal pyrogenic exotoxin-C-activated human peripheral blood mononuclear cells.** *Can J Microbiol* 1996, **42:**1104-1111.

110. Szczepanska K, Chrzanowska-Lightowlers Z, Degnan BA, Diamond AG, Robson T, Ryzewska A, Goodacre JA: **Th1-type cytokine mRNA in rheumatoid arthritis mononuclear cells induced by streptococcal pyrogenic exotoxin A.** *Rheumatology (Oxford)* 1999, **38:**1022-1024.

111. Gerlach D, Schmidt KH, Fleischer B: **Basic streptococcal superantigens (SPEX/SMEZ or SPEC) are responsible for the mitogenic activity of the so-called mitogenic factor (MF).** *FEMS Immunol Med Microbiol* 2001, **30:**209-216.

112. Proft T, Arcus VL, Handley V, Baker EN, Fraser JD: **Immunological and biochemical characterization of streptococcal pyrogenic exotoxins I and J (SPE-I and SPE-J) from Streptococcus pyogenes.** *J Immunol* 2001, **166:**6711-6719.

113. Proft T, Webb PD, Handley V, Fraser JD: **Two novel superantigens found in both group A and group C Streptococcus.** *Infect Immun* 2003, **71:**1361-1369.

114. Chiang-Ni C, Wang CH, Tsai PJ, Chuang WJ, Lin YS, Lin MT, Liu CC, Wu JJ: **Streptococcal pyrogenic exotoxin B causes mitochondria damage to polymorphonuclear cells preventing phagocytosis of group A streptococcus.** *Med Microbiol Immunol* 2006, **195:**55-63.

115. Nilsson M, Sørensen OE, Mörgelin M, Weineisen M, Sjöbring U, Herwald H: **Activation of human polymorphonuclear neutrophils by streptolysin O from Streptococcus pyogenes leads to the release of proinflammatory mediators.** *Thromb Haemost* 2006, **95:**982-990.

116. Luo YH, Chuang WJ, Wu JJ, Lin MT, Liu CC, Lin PY, Roan JN, Wong TW, Chen YL, Lin YS: **Molecular mimicry between streptococcal pyrogenic exotoxin B and endothelial cells.** *Lab Invest* 2010, **90:**1492-1506.

117. Giesbrecht K, Förmer S, Sähr A, Heeg K, Hildebrand D: **Streptococcal Pyrogenic Exotoxin A-Stimulated Monocytes Mediate Regulatory T-Cell Accumulation through PD-L1 and Kynurenine.** *Int J Mol Sci* 2019, **20**.

118. Beachey EH, Stollerman GH: **Toxic effects of streptococcal M protein on platelets and polymorphonuclear leukocytes in human blood.** *J Exp Med* 1971, **134:**351-365.

119. Valdimarsson H, Sigmundsdottir H, Jonsdottir I: **Is psoriasis induced by streptococcal superantigens and maintained by M-protein-specific T cells that cross-react with keratin?** *Clin Exp Immunol* 1997, **107 Suppl 1:**21-24.

120. Herwald H, Cramer H, Mörgelin M, Russell W, Sollenberg U, Norrby-Teglund A, Flodgaard H, Lindbom L, Björck L: **M protein, a classical bacterial virulence determinant, forms complexes with fibrinogen that induce vascular leakage.** *Cell* 2004, **116:**367-379.

121. Påhlman LI, Olin AI, Darenberg J, Mörgelin M, Kotb M, Herwald H, Norrby-Teglund A: **Soluble M1 protein of Streptococcus pyogenes triggers potent T cell activation.** *Cell Microbiol* 2008, **10:**404-414.

122. Sigurdardottir T, Björck V, Herwald H, Mörgelin M, Rutardottir S, Törnebrant J, Bodelsson M: **M1 protein from streptococcus pyogenes induces nitric oxide-mediated vascular hyporesponsiveness to phenylephrine: involvement of toll-like receptor activation.** *Shock* 2010, **34:**98-104.

123. Valderrama JA, Riestra AM, Gao NJ, LaRock CN, Gupta N, Ali SR, Hoffman HM, Ghosh P, Nizet V: **Group A streptococcal M protein activates the NLRP3 inflammasome.** *Nat Microbiol* 2017, **2:**1425-1434.

124. Kotb M, Ohnishi H, Majumdar G, Hackett S, Bryant A, Higgins G, Stevens D: **Temporal relationship of cytokine release by peripheral blood mononuclear cells stimulated by the streptococcal superantigen pep M5.** *Infect Immun* 1993, **61:**1194-1201.

125. Simpson WA, Dale JB, Beachey EH: **Cytotoxicity of the glycolipid region of streptococcal lipoteichoic acid for cultures of human heart cells.** *J Lab Clin Med* 1982, **99:**118-126.

126. Leon O, Panos C: **Cytotoxicity and inhibition of normal collagen synthesis in mouse fibroblasts by lipoteichoic acid from Streptococcus pyogenes type 12.** *Infect Immun* 1983, **40:**785-794.

127. Tomlinson K, Leon O, Panos C: **Morphological changes and pathology of mouse glomeruli infected with a streptococcal L-form or exposed to lipoteichoic acid.** *Infect Immun* 1983, **42:**1144-1151.

128. Müller-Alouf H, Alouf JE, Gerlach D, Ozegowski JH, Fitting C, Cavaillon JM: **Comparative study of cytokine release by human peripheral blood mononuclear cells stimulated with Streptococcus pyogenes superantigenic erythrogenic toxins, heat-killed streptococci, and lipopolysaccharide.** *Infect Immun* 1994, **62:**4915-4921.

129. Ganguly CL, Dale JB, Courtney HS, Beachey EH: **Tyrosine phosphorylation of a 94-kDa protein of human fibroblasts stimulated by streptococcal lipoteichoic acid.** *J Biol Chem* 1985, **260:**13342-13346.

130. Leon O, Panos C: **Effect of streptococcal lipoteichoic acid on prolyl hydroxylase activity as related to collagen formation in mouse fibroblast monolayers.** *Infect Immun* 1985, **50:**745-752.

131. Weinreb BD, Shockman GD, Beachey EH, Swift AJ, Winkelstein JA: **The ability to sensitize host cells for destruction by autologous complement is a general property of lipoteichoic acid.** *Infect Immun* 1986, **54:**494-499.

132. Levy R, Kotb M, Nagauker O, Majumdar G, Alkan M, Ofek I, Beachey EH: **Stimulation of oxidative burst in human monocytes by lipoteichoic acids.** *Infect Immun* 1990, **58:**566-568.

133. Keller R, Fischer W, Keist R, Bassetti S: **Macrophage response to bacteria: induction of marked secretory and cellular activities by lipoteichoic acids.** *Infect Immun* 1992, **60:**3664-3672.

134. Lichtman SN, Wang J, Schwab JH, Lemasters JJ: **Comparison of peptidoglycan-polysaccharide and lipopolysaccharide stimulation of Kupffer cells to produce tumor necrosis factor and interleukin-1.** *Hepatology* 1994, **19:**1013-1022.

135. Murr C, Widner B, Gerlach D, Werner-Felmayer G, Dierich MP, Wachter H, Fuchs D: **Streptococcal erythrogenic toxins induce tryptophan degradation in human peripheral blood mononuclear cells.** *Int Arch Allergy Immunol* 1997, **114:**224-228.

136. Okamoto M, Ohe G, Oshikawa T, Nishikawa H, Furuichi S, Yoshida H, Matsuno T, Saito M, Sato M: **Induction of Th1-type cytokines by lipoteichoic acid-related preparation isolated from OK-432, a penicillin-killed streptococcal agent.** *Immunopharmacology* 2000, **49:**363-376.

137. Maudsdotter L, Jonsson H, Roos S, Jonsson AB: **Lactobacilli reduce cell cytotoxicity caused by Streptococcus pyogenes by producing lactic acid that degrades the toxic component lipoteichoic acid.** *Antimicrob Agents Chemother* 2011, **55:**1622-1628.

138. Buslau M, Kappus R, Gerlach D, Köhler W, Diehl S, Holzmann H: **Streptococcal and staphylococcal superantigens (ETA, SEB): presentation by human epidermal cells and induction of autologous T cell proliferation in vitro.** *Acta Derm Venereol* 1993, **73:**94-96.

139. Michos A, Gryllos I, Håkansson A, Srivastava A, Kokkotou E, Wessels MR: **Enhancement of streptolysin O activity and intrinsic cytotoxic effects of the group A streptococcal toxin, NAD-glycohydrolase.** *J Biol Chem* 2006, **281:**8216-8223.

140. Sharma O, O'Seaghdha M, Velarde JJ, Wessels MR: **NAD+-Glycohydrolase Promotes Intracellular Survival of Group A Streptococcus.** *PLoS Pathog* 2016, **12:**e1005468.

141. Chaussee MS, Ajdic D, Ferretti JJ: **The rgg gene of Streptococcus pyogenes NZ131 positively influences extracellular SPE B production.** *Infect Immun* 1999, **67:**1715-1722.

142. Eraso JM, Kachroo P, Olsen RJ, Beres SB, Zhu L, Badu T, Shannon S, Cantu CC, Saavedra MO, Kubiak SL, et al: **Genetic heterogeneity of the Spy1336/R28-Spy1337 virulence axis in Streptococcus pyogenes and effect on gene transcript levels and pathogenesis.** *PLoS One* 2020, **15:**e0229064.

143. Zhang S, Rahman M, Zhang S, Herwald H, Qi Z, Jeppsson B, Thorlacius H: **Streptococcal M1 protein-provoked CXC chemokine formation, neutrophil recruitment and lung damage are regulated by Rho-kinase signaling.** *J Innate Immun* 2012, **4:**399-408.

144. Viszwapriya D, Subramenium GA, Prithika U, Balamurugan K, Pandian SK: **Betulin inhibits virulence and biofilm of Streptococcus pyogenes by suppressing ropB core regulon, sagA and dltA.** *Pathog Dis* 2016, **74**.

145. Theodore TS, Calandra GB: **Streptolysin S activation by lipoteichoic acid.** *Infect Immun* 1981, **33:**326-328.

146. Federle MJ, McIver KS, Scott JR: **A response regulator that represses transcription of several virulence operons in the group A streptococcus.** *J Bacteriol* 1999, **181:**3649-3657.

147. Heath A, DiRita VJ, Barg NL, Engleberg NC: **A two-component regulatory system, CsrR-CsrS, represses expression of three Streptococcus pyogenes virulence factors, hyaluronic acid capsule, streptolysin S, and pyrogenic exotoxin B.** *Infect Immun* 1999, **67:**5298-5305.

148. Nizet V, Beall B, Bast DJ, Datta V, Kilburn L, Low DE, De Azavedo JC: **Genetic locus for streptolysin S production by group A streptococcus.** *Infect Immun* 2000, **68:**4245-4254.

149. Engleberg NC, Heath A, Miller A, Rivera C, DiRita VJ: **Spontaneous mutations in the CsrRS two-component regulatory system of Streptococcus pyogenes result in enhanced virulence in a murine model of skin and soft tissue infection.** *J Infect Dis* 2001, **183:**1043-1054.

150. Lyon WR, Madden JC, Levin JC, Stein JL, Caparon MG: **Mutation of luxS affects growth and virulence factor expression in Streptococcus pyogenes.** *Mol Microbiol* 2001, **42:**145-157.

151. Dmitriev AV, McDowell EJ, Kappeler KV, Chaussee MA, Rieck LD, Chaussee MS: **The Rgg regulator of Streptococcus pyogenes influences utilization of nonglucose carbohydrates, prophage induction, and expression of the NAD-glycohydrolase virulence operon.** *J Bacteriol* 2006, **188:**7230-7241.

152. Ma Y, Bryant AE, Salmi DB, McIndoo E, Stevens DL: **vfr, a novel locus affecting cysteine protease production in Streptococcus pyogenes.** *J Bacteriol* 2009, **191:**3189-3194.

153. Treviño J, Perez N, Ramirez-Peña E, Liu Z, Shelburne SA, 3rd, Musser JM, Sumby P: **CovS simultaneously activates and inhibits the CovR-mediated repression of distinct subsets of group A Streptococcus virulence factor-encoding genes.** *Infect Immun* 2009, **77:**3141-3149.

154. Liang Z, Zhang Y, Agrahari G, Chandrahas V, Glinton K, Donahue DL, Balsara RD, Ploplis VA, Castellino FJ: **A natural inactivating mutation in the CovS component of the CovRS regulatory operon in a pattern D Streptococcal pyogenes strain influences virulence-associated genes.** *J Biol Chem* 2013, **288:**6561-6573.

155. Bao Y, Liang Z, Booyjzsen C, Mayfield JA, Li Y, Lee SW, Ploplis VA, Song H, Castellino FJ: **Unique genomic arrangements in an invasive serotype M23 strain of Streptococcus pyogenes identify genes that induce hypervirulence.** *J Bacteriol* 2014, **196:**4089-4102.

156. Makthal N, Gavagan M, Do H, Olsen RJ, Musser JM, Kumaraswami M: **Structural and functional analysis of RopB: a major virulence regulator in Streptococcus pyogenes.** *Mol Microbiol* 2016, **99:**1119-1133.

157. Port GC, Cusumano ZT, Tumminello PR, Caparon MG: **SpxA1 and SpxA2 Act Coordinately To Fine-Tune Stress Responses and Virulence in Streptococcus pyogenes.** *mBio* 2017, **8**.

158. Bernard PE, Kachroo P, Zhu L, Beres SB, Eraso JM, Kajani Z, Long SW, Musser JM, Olsen RJ: **RocA Has Serotype-Specific Gene Regulatory and Pathogenesis Activities in Serotype M28 Group A Streptococcus.** *Infect Immun* 2018, **86**.

159. Brouwer S, Cork AJ, Ong CY, Barnett TC, West NP, McIver KS, Walker MJ: **Endopeptidase PepO Regulates the SpeB Cysteine Protease and Is Essential for the Virulence of Invasive M1T1 Streptococcus pyogenes.** *J Bacteriol* 2018, **200**.

160. DebRoy S, Aliaga-Tobar V, Galvez G, Arora S, Liang X, Horstmann N, Maracaja-Coutinho V, Latorre M, Hook M, Flores AR, Shelburne SA: **Genome-wide analysis of in vivo CcpA binding with and without its key co-factor HPr in the major human pathogen group A Streptococcus.** *Mol Microbiol* 2021, **115:**1207-1228.

161. Faozia S, Fahmi T, Port GC, Cho KH: **c-di-AMP-Regulated K(+) Importer KtrAB Affects Biofilm Formation, Stress Response, and SpeB Expression in Streptococcus pyogenes.** *Infect Immun* 2021, **89**.

162. Wu ZY, Campeau A, Liu CH, Gonzalez DJ, Yamaguchi M, Kawabata S, Lu CH, Lai CY, Chiu HC, Chang YC: **Unique virulence role of post-translocational chaperone PrsA in shaping Streptococcus pyogenes secretome.** *Virulence* 2021, **12:**2633-2647.

163. Terao Y, Mori Y, Yamaguchi M, Shimizu Y, Ooe K, Hamada S, Kawabata S: **Group A streptococcal cysteine protease degrades C3 (C3b) and contributes to evasion of innate immunity.** *J Biol Chem* 2008, **283:**6253-6260.

164. Schwab JH: **An intracellular hemolysin of group A Streptococci. II. Comparative properties of intracellular hemolysin, streptolysin S, and streptolysin O.** *J Bacteriol* 1956, **71:**100-107.

165. Marchlewicz BA, Duncan JL: **Properties of a hemolysin produced by group B streptococci.** *Infect Immun* 1980, **30:**805-813.

166. Müller-Alouf H, Proft T, Zollner TM, Gerlach D, Champagne E, Desreumaux P, Fitting C, Geoffroy-Fauvet C, Alouf JE, Cavaillon JM: **Pyrogenicity and cytokine-inducing properties of Streptococcus pyogenes superantigens: comparative study of streptococcal mitogenic exotoxin Z and pyrogenic exotoxin A.** *Infect Immun* 2001, **69:**4141-4145.

167. Coye LH, Collins CM: **Identification of SpyA, a novel ADP-ribosyltransferase of Streptococcus pyogenes.** *Mol Microbiol* 2004, **54:**89-98.

168. Fiedel BA, Jackson RW: **Immunogenicity of a purified and carrier-complexed streptococcal lipoteichoic acid.** *Infect Immun* 1976, **13:**1585-1590.

169. van den Broek MF, van den Berg WB, van de Putte LB, Severijnen AJ: **Streptococcal cell wall-induced arthritis and flare-up reaction in mice induced by homologous or heterologous cell walls.** *Am J Pathol* 1988, **133:**139-149.

170. van Bruggen MC, van den Broek MF, van den Berg WB: **Streptococcal cell wall-induced arthritis and adjuvant arthritis in F344----Lewis and in Lewis----F344 bone marrow chimeras.** *Cell Immunol* 1991, **136:**278-290.

171. Esser RE, Stimpson SA, Cromartie WJ, Schwab JH: **Reactivation of streptococcal cell wall-induced arthritis by homologous and heterologous cell wall polymers.** *Arthritis Rheum* 1985, **28:**1402-1411.
